# TPGS-b-PBAE Copolymer-Based Polyplex Nanoparticles for Gene Delivery and Transfection In Vivo and In Vitro

**DOI:** 10.3390/pharmaceutics16020213

**Published:** 2024-01-31

**Authors:** Jiahui Ding, Handan Zhang, Tianli Dai, Xueqin Gao, Zhongyuan Yin, Qiong Wang, Mengqi Long, Songwei Tan

**Affiliations:** 1 School of Pharmacy, Tongji Medical College, Huazhong University of Science and Technology, Wuhan 430030, China; m201975433@alumni.hust.edu.cn (J.D.); m202175567@hust.edu.cn (H.Z.);; 2 Department of Pharmacy, Union Hospital, Tongji Medical College, Huazhong University of Science and Technology, Wuhan 430022, China; 3 Cancer Center, Union Hospital, Tongji Medical College, Huazhong University of Science and Technology, Wuhan 430022, Chinawdyxywq@hust.edu.cn (Q.W.); 4 Department of Otolaryngology, The Fifth Affiliated Hospital of Sun Yat-sen University, Meihua 52nd Road, Xiangzhou District, Zhuhai 510009, China

**Keywords:** poly (β-amino ester), gene transfection, D-α-tocopherol polyethylene glycol succinate, copolymer, CRISPR/cas9 gene editing, gene therapy

## Abstract

Poly (β-amino ester) (PBAE) is an exceptional non-viral vector that is widely used in gene delivery, owing to its exceptional biocompatibility, easy synthesis, and cost-effectiveness. However, it carries a high surface positive charge that may cause cytotoxicity. Therefore, hydrophilic d-α-tocopherol polyethylene glycol succinate (TPGS) was copolymerised with PBAE to increase the biocompatibility and to decrease the potential cytotoxicity of the cationic polymer-DNA plasmid polyplex nanoparticles (NPs) formed through electrostatic forces between the polymer and DNA. TPGS-b-PBAE (TBP) copolymers with varying feeding molar ratios were synthesised to obtain products of different molecular weights. Their gene transfection efficiency was subsequently evaluated in HEK 293T cells using green fluorescent protein plasmid (GFP) as the model because free GFP is unable to easily pass through the cell membrane and then express as a protein. The particle size, ζ-potential, and morphology of the TBP2-GFP polyplex NPs were characterised, and plasmid incorporation was confirmed through gel retardation assays. The TBP2-GFP polyplex NPs effectively transfected multiple cells with low cytotoxicity, including HEK 293T, HeLa, Me180, SiHa, SCC-7 and C666-1 cells. We constructed a *MUC2* (Mucin2)-targeting CRISPR/cas9 gene editing system in HEK 293T cells, with gene disruption supported by oligodeoxynucleotide (ODN) insertion in vitro. Additionally, we developed an *LMP1* (latent membrane protein 1)-targeting CRISPR/cas9 gene editing system in LMP1-overexpressing SCC7 cells, which was designed to cleave fragments expressing the LMP1 protein (related to Epstein–Barr virus infection) and thus to inhibit the growth of the cells in vivo. As evidenced by in vitro and in vivo experiments, this system has great potential for gene therapy applications.

## 1. Introduction

Gene therapy is an advantageous approach for addressing several illnesses, including malignant tumours, genetic diseases, infectious diseases, and cardiovascular diseases [1,2,3,4,5,6]. Some cancers, such as cervical cancer and nasopharyngeal carcinoma, are closely related to human papillomavirus (HPV) and Epstein–Barr virus (EBV) infections [7,8]. The integrated genes of these viruses in the human genome produce some onco-proteins such as E6, E7 (for HPV) and LMP1 (for EBV), which are closely related to the occurrence and development of cancer and are effective targets for gene therapy of these two cancers. One of the bottlenecks in gene therapy is the use of effective gene vectors, which can be classified as viral (such as retroviruses, lentiviruses, and adenoviruses) or non-viral (such as liposomes and cationic polymers) [2,9,10,11,12,13,14,15]. Although viral vectors have a high transfection efficiency, their safety remains a concern [16,17]. Rhesus monkeys transplanted with hematopoietic stem and progenitor cells (HSPCs) and treated with lentiviral vectors exhibited abnormal proliferation of haematopoietic cells that affected the erythroid, bone marrow, and megakaryocyte lineages [18]. Moreover, multiple genes were overexpressed and abnormally spliced. Thus, the link between clonal expansion induced by lentiviral insertion and clinically abnormal transformation after the transduction of normal primate or human HSPCs was elucidated, demonstrating the potential carcinogenicity of viral vectors. AAV-based gene therapy may induce cancer, and the U.S. Food and Drug Administration (FDA) placed a hold on some AAV-based clinical trials due to safety concerns [17]. Furthermore, the low gene loading capacity and complex packaging process of viral vectors result in a considerably elevated cost of gene therapy, thereby severely limiting their application.

Gene vectors based on cationic polymers have numerous advantages compared with viral vectors, including high safety and low costs, making them an excellent choice for gene delivery [19,20]. The gold standard of cationic polymers in gene transfection; polyethylenimine(PEI) has been widely used in many cases including clinical trials, and thus is applied as a common control of cationic polymeric gene vectors. Poly (β-amino ester) (PBAE) is also a cationic polymer that was first synthesised through the Michael addition of acrylate and amine and was utilised as a gene vector by Langer’s group [21,22,23]. It has a flexible structure and is relatively easy to synthesise. The main chain can become cationic upon exposure to an acidic environment through the amino groups, enabling PBAE to be electrostatically compressed with the negatively charged phosphate groups of nucleic acids [21,24,25]. Furthermore, ester bonds can be degraded under physiological conditions, which explains the excellent biocompatibility of PBAE. The PBAE system carries a relatively high surface positive charge that may lower its biocompatibility and cause cytotoxicity due to the cationic nature of the polymer. Therefore, additional structural optimisation is required to enhance its gene delivery performance.

Modifying the surface positive charge of the resulting polyplex nanoparticles (NPs) with hydrophilic polymers, such as polyethylene glycol (PEG), can reduce the cytotoxicity of cationic polymer-based gene vectors [9,26]. D-α-tocopherol polyethylene glycol succinate (TPGS) is a water-soluble derivative of natural vitamin E. It is formed by the esterification of vitamin E succinate and PEG1000. This compound has been approved by the Chinese National Medical Products Administration and the United States Food and Drug Administration Agency as a pharmaceutical excipient and has been used as a hydrophilic section in many drug delivery systems [27]. Therefore, TPGS was copolymerised with a PBAE system to improve its hydrophilicity and decrease cytotoxicity in this study. The impact of monomer and macromonomer feeding ratios on TPGS-b-PBAE (TBP) chemical structure, molecular weight, and transfection efficiency was initially examined. The transfection efficiency and cytotoxicity of TBP2-GFP plasmid polyplex NPs were evaluated in various cell lines including tool cells (293T), a cervical cancer cell line (HeLa, Me180 and SiHa), and a nasopharyngeal carcinoma cell line (C666-1 and SCC-7). We utilized TBP2 to establish a CRISPR/cas9 gene editing system for targeting the model gene, *MUC2*, in 293T cells [28]. The editing efficiency was confirmed through the subsequent insertion of oligodeoxynucleotide (ODN). Afterwards, we set up a CRISPR/cas9 gene editing system that focuses on the *LMP1* fragment in LMP1 (latent membrane protein 1, related to Epstein–Barr virus (EBV) infection)-overexpressed SCC7 cells (SCC7) so as to simulate an EBV-positive subcloned cell line (C666-1) of human nasopharyngeal carcinoma cell lines [29]; we also conducted pharmacodynamic experiments on mice. The findings of this study could substantially improve the applicability of non-viral vectors in gene therapy.

## 2. Materials and Methods

### 2.1. Materials, Cells, and Animals

Briefly, 1,4-butanediol diacrylate (BDD), acryloyl chloride, and 5-amino-1-pentanol (AP) were purchased from TCI (Shanghai, China); 1-(3-aminopropyl)-4-methyl piperazine (AMP) was obtained from Alfa Aesar (Shanghai, China). TPGS was purchased from Sigma-Aldrich (St. Louis, MO, USA), and 3-(4,5-Dimethylthiazol-2-yl)-2,5-diphenyltetrazolium bromide (MTT) was purchased from Biosharp (Hefei, China). Analytical-grade solvents, including N, N-dimethylformamide (DMF), ethanol, triethylamine (TEA), and diethyl ether, were procured from Sinopharm (Beijing, China). The CRISPR plasmids MUC2P and EBV3, which were designed based on spCas9 and frCas9 [30,31], as well as SCC7L cells were purchased from Generulor Co., Ltd. Bio-X Lab (Zhuhai, China)

HEK 293T, HeLa, Me180 and SiHa cells were purchased from kinlogix Co., Ltd. (Guangzhou, China), SCC-7 cells were purchased from OTWO Biotech Co., Ltd. (Shenzhen, China), and C666-1 cells were purchased from Procell Life Science & Technology Co., Ltd. (Wuhan, China). HEK 293T, HeLa, Me180, and SiHa cells were cultured in Dulbecco’s modified eagle medium (DMEM) complete medium. C666-1 and SCC7L cells were cultured in RPMI 1640 complete medium. The cell incubator was set 37 °C and 5% CO_2_, with saturated humidity. When the cells reached a density of approximately 80%, they were digested with trypsin and separated at a ratio of 1:3.

BALB/c-nu (SPF, 17–19 g, male) and KM mice (SPF, 17–19 g, male) were raised at the SPF isolation package laboratory in the Animal Center of Tongji Medical College, Huazhong University of Science and Technology (Wuhan, China). The research was approved by the Experimental Animal Ethics Committee of Tongji Medical College of Huazhong University of Science and Technology.

### 2.2. Synthesis and Characterisation of TPGS-b-PBAE

**Figure 1:**
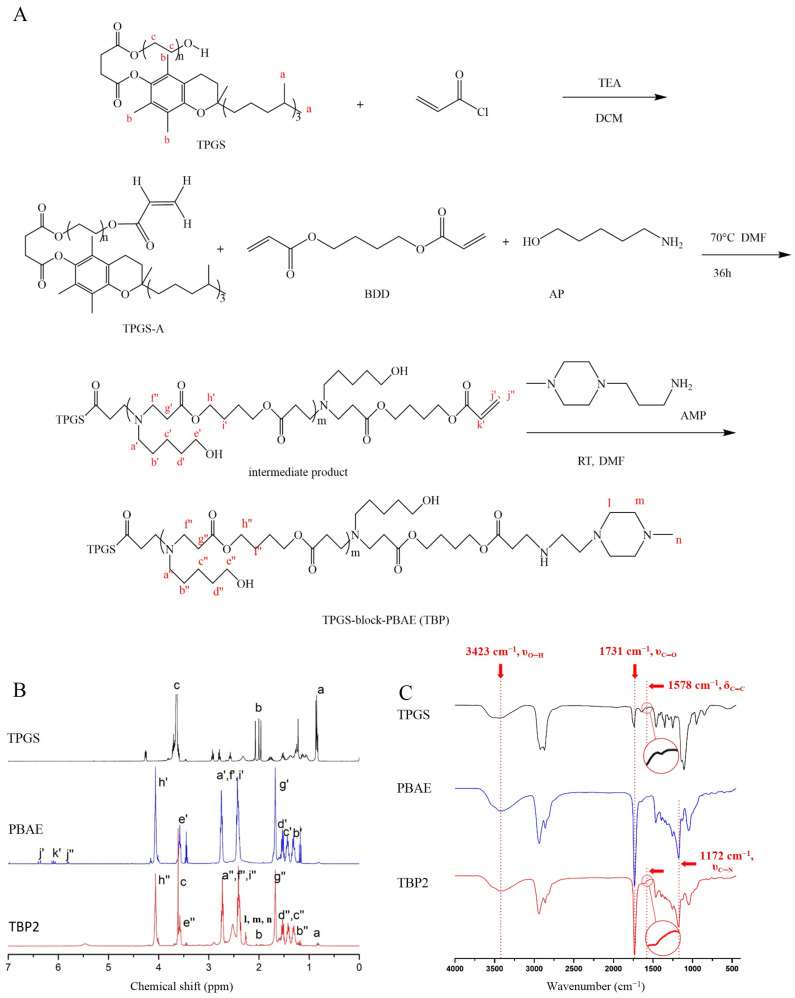
Synthesis and characteristics of TBP: (**A**) synthesis scheme, (**B**) ^1^H-NMR spectra of TPGS, PBAE and TBP2, and (**C**) FTIR spectra of TPGS, PBAE and TBP2.

As shown in Figure 1A, TPGS (1513 mg, 1 mM) and acryloyl chloride (136 mg, 1.5 mM) were reacted in 4 mL of anhydrous DMF for 12 h, with TEA (152 mg, 1.5 mM) as the acid-binding agent. Following centrifugation, the supernatant was dialysed against water in a dialysis bag (MWCO 1000) for 12 h, with the outer phase changed every 4 h. TPGS-A was obtained by subsequent lyophilisation. Different molar ratios of BDD, AP, and TPGS-A were co-dissolved in DMF, as shown in Table 1. After stirring at 90 °C for 36 h, the Michael addition polymerisation was terminated and the mixture poured into six-fold volume of diethyl ether to collect the precipitant. The samples were washed twice with diethyl ether and dried under reduced-pressure distillation at room temperature for 12 h to obtain an intermediate product (denoted as TBP1=, TBP2= and TBP3=). Finally, the intermediate product was reacted with five-fold AMP. The final product was purified using the same method as that used for the intermediate product after the reaction was allowed to proceed at room temperature for 24 h. PBAE was synthesised as shown in Figure S1 and purified using the same method as that used for TBP. Fluorescent molecule (Ce6)-labelled PBAE (Ce6-PBAE) was synthesized according to our previous work using 1,3-diaminopropane to replace AMP and then conjugated with Ce6 via EDC/NHS [11].

The structures of the products in each step were characterised using ^1^H-NMR spectroscopy (Bruker AVANCE III 400 MHz NMR spectrometer, CDCl_3_) and Fourier transform infrared spectroscopy (FTIR, PerkinElmer Spectrum Two, 32 replicate scan settings). The molecular weights of TBP1, TBP2, and TBP3 were determined by gel permeation chromatography (GPC, Waters, mobile phase: DMF, standard: narrow-disperse polystyrene).

### 2.3. Preparation and Characterisation of the TBP2-GFP Polyplex NPs

TBP was dissolved in an acidic buffer (pH 5.0) to prepare the TBP2-GFP polyplex NPs. Then, the GFP or *MUC2* targeting the CRISPR/cas9 plasmid (denoted as MUC2P) solution was added to the TBP2 solution at different mass ratios (mTBP2/m plasmid) and incubated for 20 min at room temperature. The plasmid compression capacity of TBP2 was assessed through agarose gel electrophoresis using different TBP2/GFP mass ratios (5:1, 10:1, 20:1, 30:1, 50:1, 60:1, 75:1, and 100:1). Then, the polyplex NPs solution was treated with heparin (12.5 mg/mL) for 30 min to observe the release of the GFP plasmid. The diameter, ζ-potential, and morphology of polyplex NPs were measured using dynamic light scattering (DLS, ZetaPALs, Brookhaven, GA, USA) and transmission electron microscopy (TEM, JEM-1230, Japan). TBP2-GFP polyplex NPs were prepared in the same way.

### 2.4. MTT Assays

Cells were seeded in 96-well plates at a density of approximately 7000 cells per well. The cells were grown to a density of approximately 80%. The medium was exchanged with different concentrations (0.5, 1, 2.5, 5, 10, 15, 30, 50, 75, 100, and 150 µg/mL) of TBP2/TBP2-GFP solutions dispersed in serum-free DMEM/1640. Each concentration was detected five times to minimise errors. After 36 h incubation, 10 µL MTT (5 mg/mL) was added to each well, followed by another 3 h incubation. Next, the medium in the plate was carefully removed and 150 µL DMSO was added. Then, the plate was placed in a 37 °C incubator for 15 min and protected from light before detection on a microplate reader (Thermo Fisher Scientific, Waltham, MA, USA) at a wavelength of 490 nm.

### 2.5. Transfection Efficiency of the TBP2-GFP Polyplex NPs to Different Cell Lines

TBP2-GFP polyplex NPs with different mass ratios (30:1, 50:1, 75:1, and 100:1) were prepared as described above. The cells were seeded into six-well plates at a density of 3 × 10^5^ cells per well. Nanoparticle solutions were added to the corresponding wells at different mass ratios (30, 50, 75, and 100). Polyplex NPs of GFP and PEI (25 kD; Aladdin, China) were prepared and added simultaneously as positive controls. The negative control consisted of cells that underwent the same operation but without the addition of the drug. The plates were then gently swung to separate the polyplex NPs. After 36 h, images of the cells were recorded using an inverted fluorescence microscope with both white and blue light channels. Thereafter, the cells were washed with PBS, digested, collected, suspended in 400 μL PBS, and analysed using flow cytometry (Accuri C6, BD, Franklin Lakes, NJ, USA) to quantitatively detect their transfection efficiency.

### 2.6. Targeted Disruption of MUC2 by the TBP2-CRISPR/cas9 Polyplex NPs in 293T Cells

CRISPR/cas9 systems (spCas9) targeting *MUC2* in the human genome and oligodeoxynucleotide (ODN) were constructed using Generulor (Zhuhai, China) and verified by Sanger sequencing. The sgRNA sequence was GGGGCACCTAGAGTGACCAG and the PAM sequence was AGG. The plasmid was amplified in Bacillus coli and extracted using a DNA extraction kit (ZS-M11002S). Plasmid concentration was measured using Nanodrop (Thermo). Next, 293T cells were transfected with TBP2-MUC2P/ODN polyplex NPs at a mass ratio of 50:1. The genomes of 293T cells were extracted using a DNA extraction kit. The forward and reverse primers for *MUC2* (F, R) and ODN (F, R) were designed to detect gene disruption and ODN insertion. The primer sequences are listed in Table S1. The samples were subjected to agarose gel electrophoresis to detect the gene-editing ability of the TBP2-MUC2P polyplex NPs after PCR amplification of target sequences.

### 2.7. In Vivo Biodistribution of TBP2-GFP Polyplex NPs

The in vivo bio-distribution of TBP2-GFP polyplex NPs was investigated in SCC7L xenograft tumour-bearing mice. A sufficient number of SCC7L cells were cultured, digested, and washed twice with sterile PBS. The cells were subsequently resuspended in sterile PBS. Next, 5 × 10^6^ cells were injected into the armpit of each nude mouse. When the subcutaneous tumour volume of each nude mouse reached 50–100 mm^3^, mice were randomly assigned to one of three groups: Free Ce6, TBP2-GFP, or PEI-GFP, with three nude mice in each group. Each mouse received a peritumoral injection of 15 μg of plasmid (TBP2/GFP at a mass ratio of 50:1 or PEI/GFP at a mass ratio of 4.8:1), and the same small dose of TBP2-Ce6 was used to create NPs for the fluorescence intensity quantification. Mice in the Free Ce6 group were administered a free Ce6 solution with uniform fluorescence intensity. The drug solution was injected into the tumour site. All mice were anaesthetised with isoflurane gas 0, 0.5, 1, 2, 4, 8, 12, and 24 h after injection, and the distribution of the drug at the tumour site was observed. A small animal in vivo imaging system was used to determine the fluorescence intensity of Ce6 at the tumour site in the 700 nm channel (Pearl Imager, LICOR, Lincoln, NE, USA). This enabled the observation of drug retention in the tumour and its distribution in vivo. Nude mice were euthanised 24 h after injection. Then, their major organs (heart, liver, spleen, lungs, and kidneys) and the tumour were dissected. The distribution of drugs in different organs was determined by measuring the fluorescence intensity of Ce6 in each excised organ, using a small animal in vivo imaging system.

### 2.8. In Vivo Gene Editing and Safety Evaluation

To evaluate the in vivo gene delivery capability of TBP2, a CRISPR/cas9 plasmid targeting *LMP1* based on frCas9 (EBV3) was built (sgRNA sequence: GGCCTTCTTTCCTTGTCCTTAC, PAM sequence: TGTA). The gene editing of a TBP2-EBV3 polyplex was evaluated in a SCC7L cell model (KM mice). The preparation and injection of cells and dosage of plasmids for each mouse were the same as described in Section 2.7, with slight modifications. When the tumour volume of each mouse reached 50–100 mm^3^, the mice were randomly divided into three groups: PBS, TBP2-EBV3, and PEI-EBV3, with five mice in each group. After grouping, drugs were injected around the tumour site every two days, and the tumour size (measuring the length and width of the tumour with a vernier caliper) and mouse weight were measured daily. The mice were injected four times. After the fourth injection, they were euthanised. Then, their major organs (heart, liver, spleen, lungs, and kidneys) and tumours were dissected and weighed. The tumours were photographed according to their respective groups. Tumour volume was calculated as follows: volume = length × width × width/2. The tumour inhibition rate (TIR) was calculated based on the tumour weight (TW) as follows: TIR (%) = [1 − (TW of experimental group)/(TW of control group)] × 100%. Portions of major organs and tumours from each group were removed, washed with PBS, fixed in 4% paraformaldehyde, processed for routine paraffin embedding, and subjected to haematoxylin and eosin (H&E) staining. Biochemical tests for alanine aminotransferase (ALT) and blood urea nitrogen (BUN) were performed on three randomly selected mice from each group to assess liver and kidney function.

### 2.9. Statistical Analysis

All quantitative data are represented as mean ± SD from at least three parallel measurements. Statistical analysis was performed using a one-way ANOVA or Student’s *t*-test in GraphPad Prism software (version 7.00), with *p* < 0.05 indicating significant difference.

## 3. Results and Discussion

### 3.1. Synthesis and Characterisation of TBP (TBP)

TPGS-A was obtained through an acylation reaction between TPGS and acyl chloride, followed by two Michael addition steps. As illustrated in Figure S2, the uncapped intermediate products of TBP1=, TBP2=, and TBP3= with acrylate-amino ratios of 1:0.9, 1:1, and 1:1.1, respectively, exhibited similar ^1^H NMR spectra, except for the varying end-group (acrylate) content. The peak of the ethylene band appeared between 5.80 and 6.50 ppm (j′, j″, k′) and appeared more clearly in TBP1= compared to TBP2= and TBP3=. The peak of the ethylene band disappeared after the reaction with AMP (Figure S3), revealing the successful terminal group modification. In the spectra of final products, such as TBP2 (Figure 1B), typical chemical shifts of TPGS (0.86(a), 3.61 ppm(c)), BDD (4.11 ppm (h′, h″), −OCOCH_2_−), and AP (3.52 ppm (e′, e″), and CH_2_OH) units were found in TBP, indicating the successful synthesis of TBP2. All the other chemical shift signals were observed in the spectra. These results demonstrate the successful synthesis of TBP.

The FT-IR spectra of TBP1, TBP2, and TBP3 were similar (Figure 1C and Figure S4) because they possess identical functional groups. As illustrated in Figure 1C, TBP2 exhibited the presence of TPGS (1578 cm^−1^, δ_C=C_ in benzene ring), and PBAE (1172 cm^−1^, ν_C-N_, 1731 cm^−1^, ν_C=O_, and 3423 cm^−1^, ν_O-H_). The GPC results (Table 1 and Figure S5) revealed that the weight-average molecular weight (Mw) of TBP1, TBP2, and TBP3 was 22,110, 39,340, and 40,330 g/mol, respectively. As reported, the molecular weight of PBAE increases as the ratio of monomers nears stoichiometric equivalence, and an excess of amine or diacrylate monomer results in amine- or acrylate-terminated chains, respectively [32]. In this reaction, TPGS-A will react with equal AP, so the molar rations of BDD and AP for TBP1, TBP2 and TBP3 were 95:85, 95:90 and 95:100, respectively. Impurities in the monomers (analytical purity) may cause slight shift in molecular weight, too. As a result, the average molecular weights of TBP2 and TBP3 were larger than that of TBP1, while they were close to each other for TBP2 and TBP3.

### 3.2. Transfection Efficiency of TBP1, TBP2, and TBP3 Composite GFP Polyplex NPs on 293T Cell Lines

**Figure 2:**
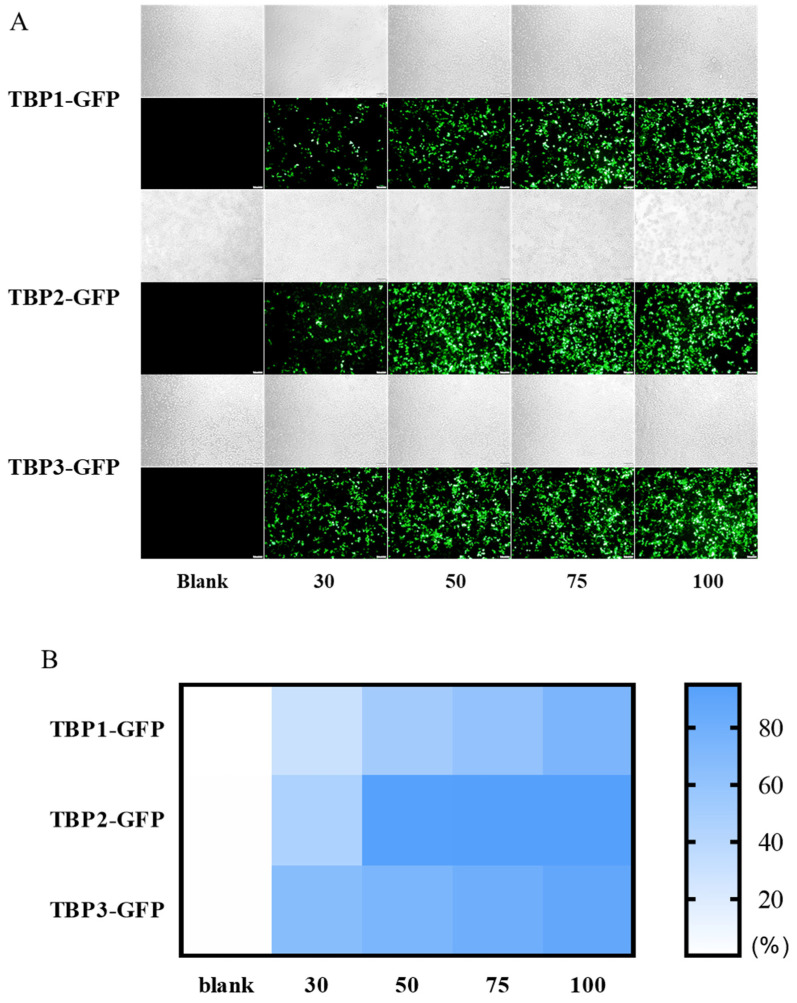
Representative fluorescence microscopy (**A**) and quantitative cytometric analysis of a transfection efficiency heat map (**B**) of 293T with TBP1, TBP2, TBP3 at a mass ratio of 30:1, 50:1, 75:1, 100:1. Bar = 100 μm.

The HEK 293T cell line was used as the cell model to evaluate the gene transfection capability of TBP1, TBP2, and TBP3. As shown in Figure 2A, the fluorescence intensity of the three TBP groups increased with an increasing mass ratio. The TBP2-GFP group exhibited the strongest fluorescence intensity. The quantitative transfection efficiency was measured by flow cytometry, as shown in Figure 2B and Figure S6. TBP3-GFP exhibited superior performance at a mass ratio of 30:1, with a transfection efficiency of 66.0%. In contrast, TBP2-GFP displayed a higher transfection efficiency than that of the other two groups at all the mass ratios. A maximum transfection efficiency of 93.4% was achieved at a mass ratio of 50:1, which was significantly higher than that of TBP1 (100:1, 73.0%) and TBP3 (100:1, 87.1%). These results suggest that the transfection efficiency of TBP is influenced by molecular weight and the end group. For TBP1 and TBP2, which have the same AMP end group, a higher molecular weight improved transfection efficiency (39,340 Da vs. 22,110 Da for TBP2 and TBP1). The AMP end group in TBP2 exhibited a stronger transfection capability than the that of AP-ended TBP3 when TBP2 and TBP3 had the same molecular weight. It has been widely reported that the end group of PBAE will impact the physicochemical properties and gene transfection efficiency. Compared to AP (monoamine)-ended TBP3, the AMP end group in TBP2 may enhance the DNA binding strength and improve the interaction with cell membranes, resulting in a higher transfection efficiency [33]. Furthermore, the transfection efficiency was enhanced by a higher molecular weight, as observed in the comparison between TBP2 (39,340 Da) and TBP1 (22,110 Da). TBP2 was subsequently selected for further experiments because of its superior transfection performance in terms of dosage and transfection efficiency.

### 3.3. Characterisation of the TBP2-GFP Polyplex NPs

**Figure 3:**
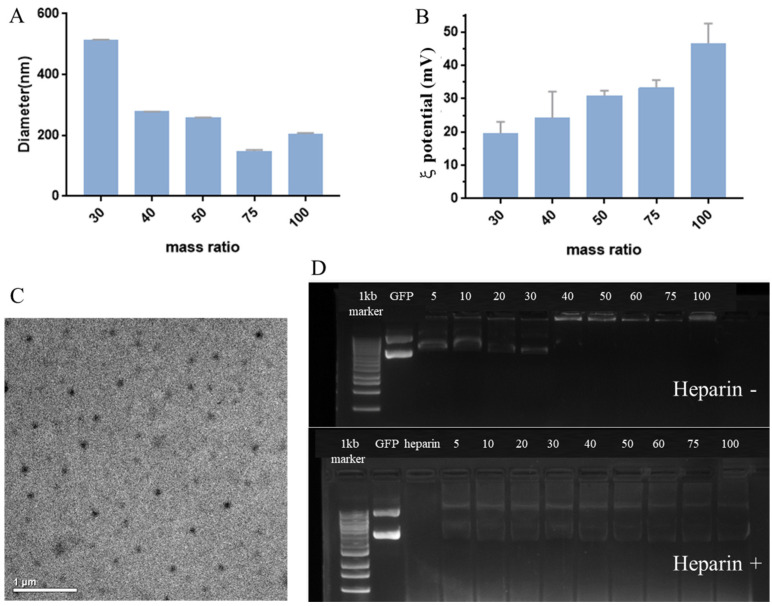
Characterization of TBP2-GFP polyplex NPs: particle size (**A**) and ζ-potential (**B**) of NPs at different mass ratios (DLS, the data represent the average value of three tests), TEM image (**C**) at a mass ratio of 75:1 (scale bar: 1 µm), and agarose gel electrophoresis (**D**) pattern of NPs at different mass ratios with or without the existence of heparin (12.5 mg/mL).

The particle diameters (Figure 3A and Figure S7) and ζ-potential (Figure 3B) of the TBP2-GFP polyplex NPs measured using DLS at different mass ratios (30:1, 40:1, 50:1, 75:1) showed that the particle size of the TBP2-GFP polyplex NPs decreased and the ζ-potential increased as the mass ratio increased, owing to the change of net electrostatic repulsive forces. Compared to PBAE-GFP polyplex NPs, the ζ-potential of the TBP2-GFP polyplex NPs decreased owing to the existence of TGPS in TBP2 (Figure S8A). The particle diameter of the composite polyplex NPs was the smallest (approximately 140 nm) at 75:1. It should be noted that the size of TBP2-GFP at a mass ratio of 100:1 was larger than that at 75:1, which may be caused by the excessive polymer aggregated into the polymer/DNA polyplex [34]. The TEM images (Figure 3C) of the TBP2-GFP polyplex NPs at 75:1 showed that the composite polyplex NPs were spherical particles with uniform distribution and good dispersibility. Furthermore, their particle size was approximately 60–100 nm, which was smaller than that of the composite polyplex NPs measured using DLS, owing to dehydration of the polyplex NPs. We further evaluated the stability of TBP2-GFP polyplex NPs and PBAE-GFP polyplex NPs. During the 8 h storage in DMEM, the particle size of PBAE-GFP polyplex NPs increased slightly after 4 h, whereas the particle size of TBP2-GFP polyplex NPs changed significantly after 8 h, indicating improved stability compared to PBAE-GFP polyplex NPs (Figure S8B) owing to the existence of the hydrophilic PEG segment of TPGS.

Gel electrophoresis (Figure 3D) demonstrated that TBP2 can encapsulate the GFP plasmid. The DNA band disappeared as the mass ratio increased. When the mass ratio was 30:1, there were visible DNA bands. However, this visibility disappeared when the mass ratio reached 40:1, indicating that the GFP plasmid was completely encapsulated by TBP. Because heparin is negatively charged under physiological conditions, it can compete with negatively charged plasmids and help release plasmids from the polyplex NPs. In the present study, an obvious plasmid band was observed after incubation with heparin, indirectly proving that TBP2 successfully encapsulated the plasmids.

### 3.4. In Vitro Safety Evaluation and Transfection Efficiency of the TBP2/TBP2-GFP Polyplex NPs in Multiple Cell Lines

The positively charged TBP2/TBP2-plasmids polyplex NPs may be cytotoxic to the cells and thus affect the transfection efficiency, which needed to be investigated first. The growth and proliferation of TBP2/TBP2-GFP substantially varied among diverse cell lines. In some cases, the cell viability was more than 100%; this is because TBP2/TBP2-GFP at a certain concentration is non-toxic to cells. The cell density has randomness within a certain range; this is perhaps due to the experimental operation. This phenomenon can be also found in some publications [35,36]. In the 293T, C666-1, and SCC7 cell lines, TBP2/TBP2-GFP exhibited negligible toxicity, with cell viability of ~80% at concentrations <50 μg/mL. However, there was a considerable decrease in cell viability at concentrations >75 µg/mL in the 293T cell line. For HeLa and Siha cells, the best tolerance for the free TBP2 solution with a cell viability of about 80% was found at a concentration of 75 µg/mL. Cell viability slowly decreased with the increase in TBP2/TBP2-GFP concentration in Me180, with a critical concentration ranging between 10 and 30 µg/mL. The ranking of tolerance against TBP2-GFP polyplex NPs was as follows: SiHa > C666-1 > HeLa > SCC7 ≥ 75 µg/mL > 293T ≥ 50 µg/mL > Me180, demonstrating excellent safety for application for most of the cells.

**Figure 4:**
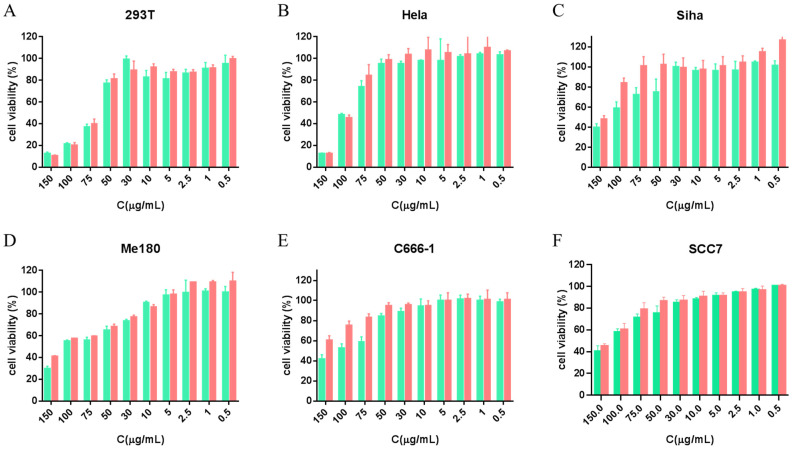
Cell viability of TBP2(green)/TBP2-GFP(red) in 293T (**A**), HeLa (**B**), SiHa (**C**), Me180 (**D**), C666-1 (**E**), and SCC7 (**F**) cells, as calculated by MTT assays.

Safety/cytotoxicity should be considered when evaluating the performance of a gene vector. As shown in Figure 4, the cytotoxicity of TBP2 was higher than that of the TBP2-GFP polyplex NPs in all cell lines. This phenomenon can be explained by the fact that positively charged TBP2 impairs normal cell function, thus affecting its growth. After TBP2 combined with a negatively charged GFP plasmid, the positively charged characteristics of the plasmid were partially neutralised, resulting in decreased cytotoxicity. The growth and proliferation of TBP2/TBP2-GFP varied considerably among diverse cell lines. In the 293T cell line, TBP2/TBP2-GFP exhibited negligible toxicity, with cell viability exceeding 80% at concentrations <50 µg/mL. However, there was a considerable decrease in cell viability at concentrations >75 µg/mL. A similar inhibition behaviour was observed in HeLa cells, with a critical concentration ranging between 75 and 100 µg/mL. Cell viability slowly decreased with the increase in TBP2/TBP2-GFP concentration in Me180, SiHa, C666-1 and SCC7 cells. Figure S9 shows the IC50 values calculated from the MTT results, which demonstrate the cytotoxicity tolerance of different cells to TBP2/TBP2-GFP. SCC7 cells exhibited the best tolerance for the free TBP2 solution (IC50 = 125.5 ± 2.3 µg/mL). The subsequent ranking of tolerance was as follows: SiHa > C666-1 > HeLa > Me180 > 293T. For the TBP2-GFP polyplex NPs solution, this tendency slightly differed from the increasing tolerance order of 293T, HeLa, Me180, SiHa, C666-1, and SCC7 cells. The IC50 of TBP2-GFP in C666-1 was 188 µg/mL, which was much higher than the 111.4 ± 2.0 µg/mL observed when cells were treated with the free TBP2 solution. For Me180, SiHa and SCC7 cells, the IC50 value also improved in the form of TBP2-GFP polyplex NPs. It can be also observed that the IC50 value of TBP2-GFP polyplex NPs (73.5 ± 1.6 µg/mL) in 293T cells was slightly higher than that of PBAE-GFP polyplex NPs (71.3 ± 0.5 µg/mL, Figure S10), which may be owing to decreased ζ-potential of TBP2-GFP compared to PBAE-GFP.

**Figure 5:**
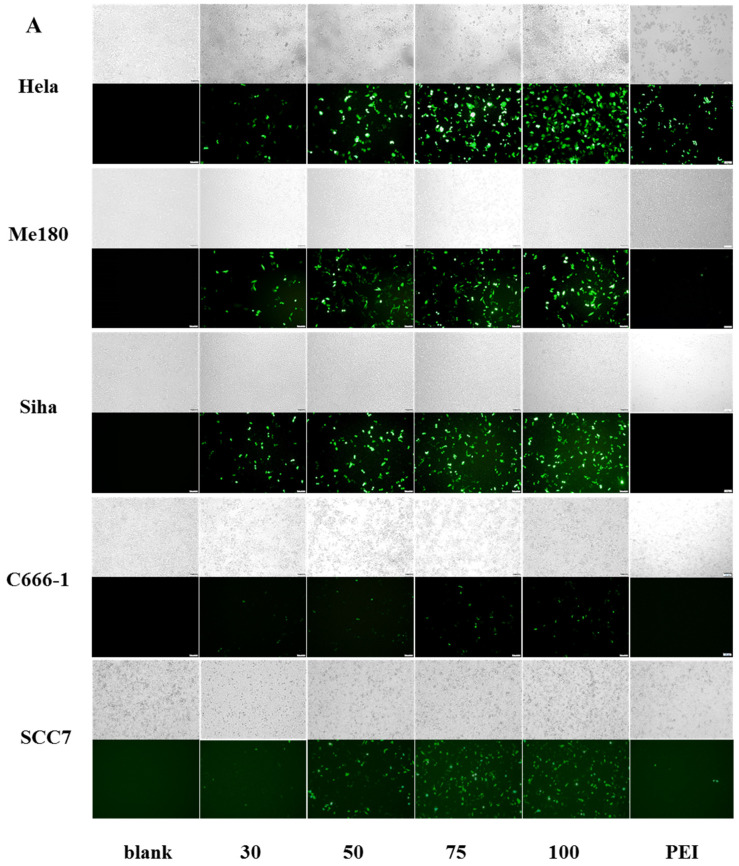

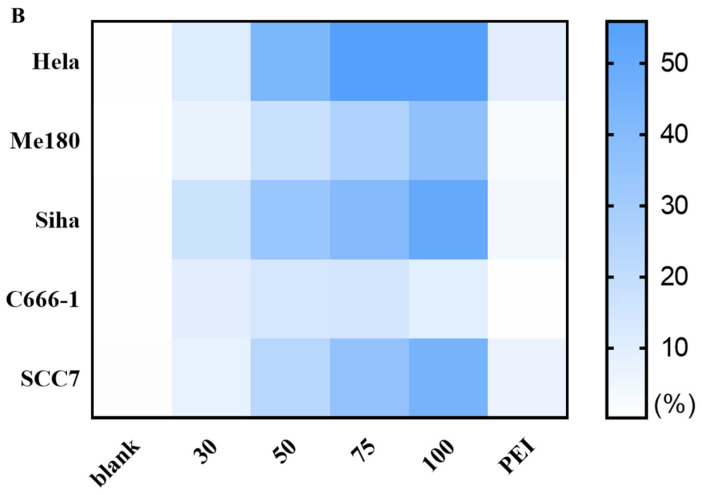
Representative fluorescence microscopy (**A**) and quantitative transfection efficiency (**B**) of transfection of HeLa, Me180, SiHa, C666-1 and SCC-7 with TBP2-GFP polyplex NPs at a mass ratio of 30:1, 50:1, 75:1, 100:1 (PEI25kD as positive control). Bar = 100 μm.

Then, fluorescence microscopy was used to assess the transfection efficiency of TBP2 in various cell lines. Figure 5A shows fluorescence microscopy images of the TBP2-GFP polyplex NPs in 293T, HeLa, Me180, SiHa, C666-1 and SCC-7 cells. Although TBP2-GFP exhibited considerable transfection efficiency in 293T cells (>90% at 50:1), the transfection efficiency in tumour cells decreased to varying degrees. The cytometry results (Figure S11 and Figure 5B) are consistent with the overall trend observed in the microscopy images. For most cell lines, such as HeLa, Me180, SiHa and SCC7, the transfection efficiency increased with an increasing mass ratio, and the optimal mass ratio for Me180, SiHa and SCC7 was 100:1, while for HeLa, it was 75:1. The highest transfection efficiency was observed at mass ratios of 50:1 and 75:1 in C666-1 cells, but the transfection efficiency was only 14%. TBP2 performed better than the commercial cationic polymeric reagent PEI25kD in all the cell lines. The maximum transfection efficiency of PEI-GFP in the cell lines was less than 10%. TBP2 successfully transfected multiple tumour cells and outperformed the commercialised PEI in most circumstances.

More importantly, the concentration of the TBP2-GFP polyplex NPs was 50, 75, 100, and 75 µg/mL at maximum transfection efficiency in 293T, HeLa, SiHa, and C666-1 cells, respectively. The viability was >80% in all four cell lines, demonstrating excellent safety for application. The viability of Me180 cells was approximately 60% when the mass ratio was 100, indicating that TBP2 may not be suitable for gene transfection in this cell line. For SCC-7 cells, the safe concentration was 75 µg/mL; the corresponding transfection efficiency was 35.5%, and thus this value was applied in the following experiments.

### 3.5. In Vitro Gene Editing of TBP2-Plasmids Polyplex NPs

**Figure 6:**
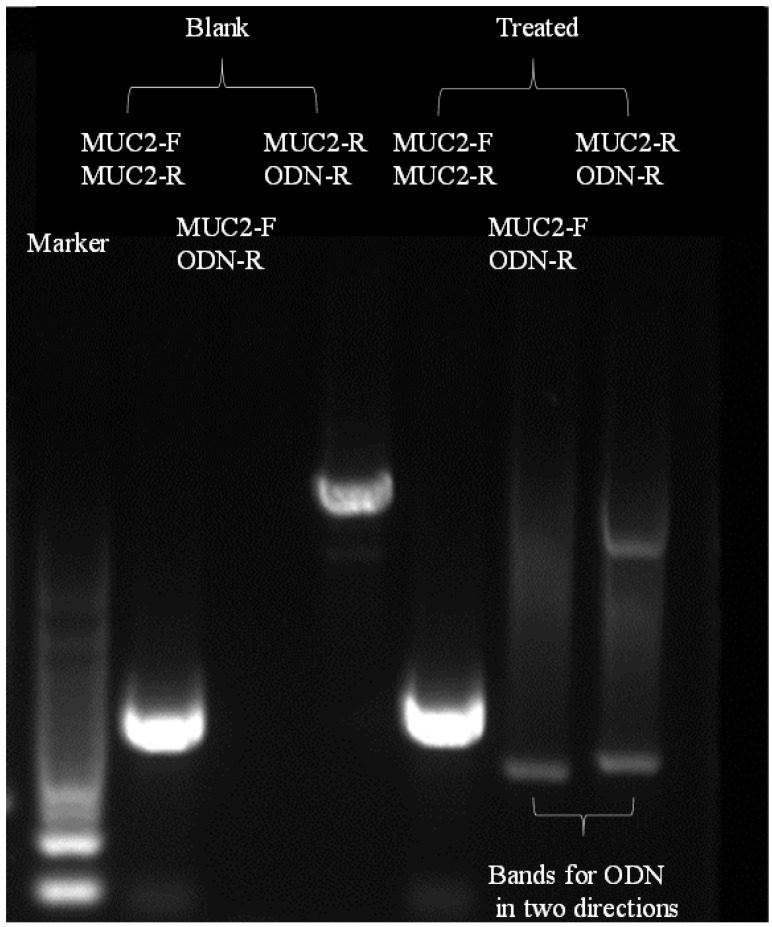
Agarose gel electrophoresis results after ODN-PCR. Bands of MUC2-F and MUC2-R were the PCR products of the primer MUC2-F and primer MUC2-R, bands of MUC2-F and ODN-R were the PCR products of the primer MUC2-F and primer ODN-R, and bands of MUC2-R and ODN-R were the PCR products of the primer MUC2-R and primer ODN-R.

It is known that 293T cells have many basic applications in gene editing. So, we first applied 293T cells as a model for investigating the potential of TBP2 to deliver the CRISPR/cas9 gene editing system through the disruption of *MUC2* function by TBP2-MUC2P polyplex NPs. The ODN was designed to investigate the feasibility of this gene editing system, as it can be inserted into the disruption site [37]. In the blank group, DNA treated with MUC2-F and MUC2-R (former and reverse primers of *MUC2*, Table S1) exhibited a clear band during agarose gel electrophoresis, indicating the presence of *MUC2* in 293T cells, as observed through ODN-PCR (Figure 6). In contrast, DNA treated with the ODN primer R in the blank group did not show bands at certain locations. However, bands appeared in the group treated with TBP2-MUC2P and the primer combination MUC2-F/ODN-R or MUC2-R/ODN-R, demonstrating that the ODN was successfully inserted into the disruption site created by the cas9 nuclease. The direction of insertion was random because both prime ODNs could amplify the sequence. These findings demonstrate that TBP2 can deliver CRISPR/cas9 systems into cells, and that sgRNA and the cas9 nuclease can take effect. This provides the basis for the application of TBP2 in gene therapy.

### 3.6. Bio-Distribution of the Polyplex NPs

**Figure 7:**
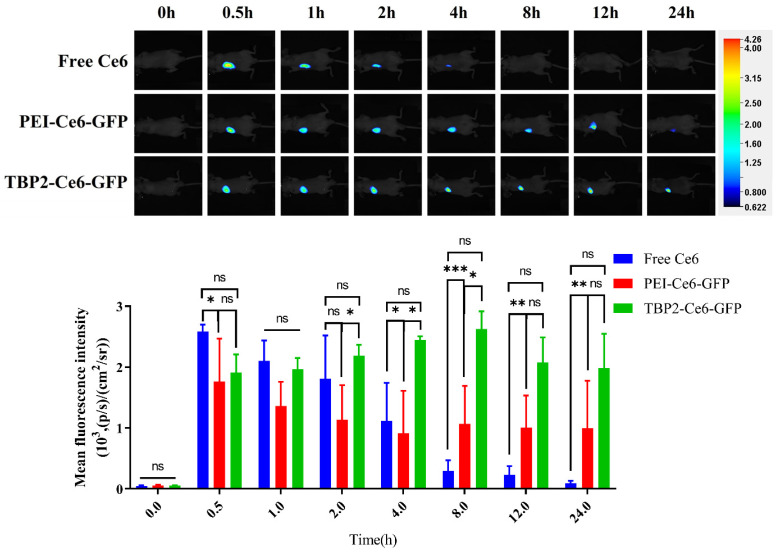
The distribution of different formulations (Free Ce6, PEI-Ce6-GFP and TBP2-Ce6-GFP) after injection over time, as monitored by living images (*n* = 3; a two-way ANOVA was used for statistical analysis; *** *p* < 0.001, ** *p* < 0.01. * *p* < 0.05; ns: no significant difference). Colour bar: fluorescence intensity [(p/s)/(cm^2^/sr)].

**Figure 8:**
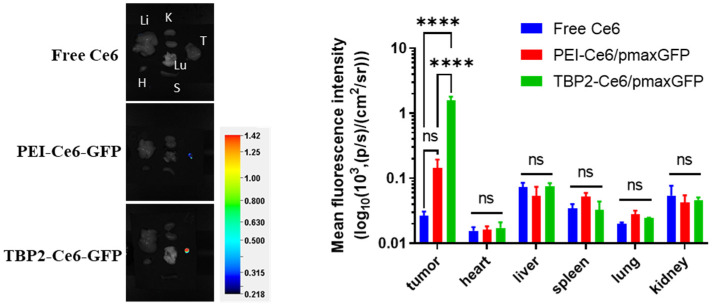
The fluorescence intensity of major organs (ex vivo, H: heart, Li: liver, K: kidneys, Lu: lung, S: spleen, T: tumor) in each group 24 h after injection of different formulations (Free Ce6, PEI-Ce6-GFP and TBP2-Ce6-GFP) (*n* = 3; a one-way ANOVA was used for statistical analysis; **** *p* < 0.0001; ns: no significant difference). Colour bar: fluorescence intensity [(p/s)/(cm^2^/sr)].

The biodistribution of TBP2-EBV3 polyplex NPs was evaluated in SCC7L-tumor bearing mice using Ce6 as a probe. After peritumoral administration with free Ce6 and Ce6-labelled polyplex NPs, it can be observed that free Ce6 was observed to be rapidly metabolised, and there was minimal retention at the tumour site after 24 h. In contrast, the TBP2-EBV3 and PEI-EBV3 groups exhibited significantly better tumour retention than the free Ce6 group did. The TBP2-EBV3 group exhibited a significantly stronger fluorescence signal than the PEI-EBV3 group did during the first 8 h, and the mean fluorescence intensity showed no difference throughout the entire experimental testing period, suggesting that TBP2-EBV3 NPs possess exceptional tumour retention capacity and have a prolonged effect (Figure 7 and Figure S12). Figure 8 and Figure S13 show the ex vivo results of the major organs in every group 24 h post injection. It is shown that only weak fluorescence was observed in the heart, liver, spleen, lungs, and kidneys in all three groups. The low distribution in major organs may be due to the positive charge of the NPs increasing the interaction with negative charged tumour cell membrane and also to the low uptake of these organs to degradations released from the tumour. The order of fluorescence intensity in the tumour tissues was as follows: TBP2-EBV3 > PEI-EBV3 > Free Ce6. This suggested that TBP2-EBV3 polyplex NPs had the most effective retention capability, with notable differences between TBP2-EBV3 and the other two groups, thus suggesting that TBP2-EBV3 is an effective gene therapy system for local tumour administration.

### 3.7. In Vivo Gene Editing and Safety Evaluation in KM Mice

**Figure 9:**
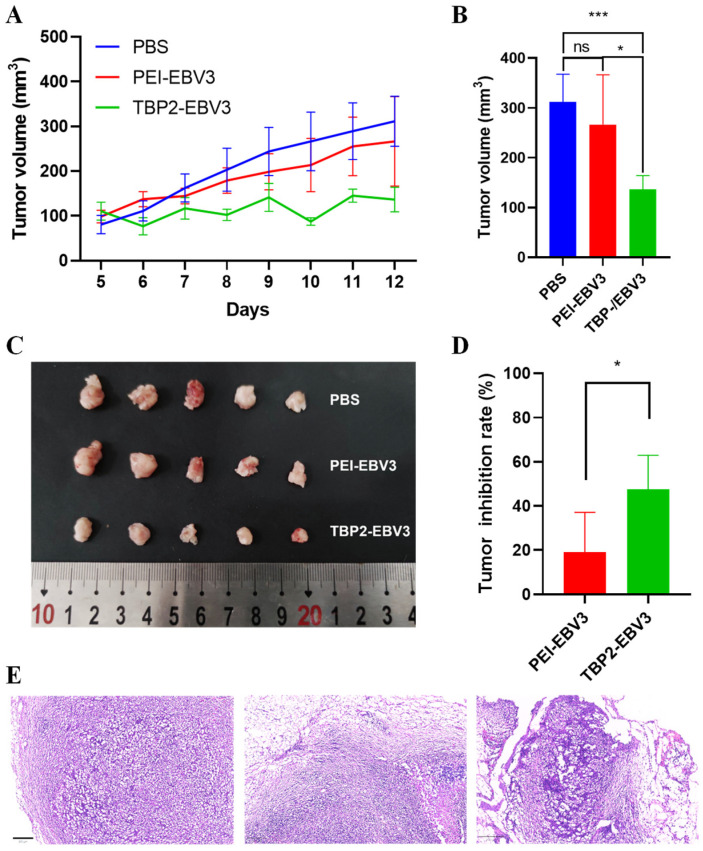
In vivo gene editing of the polyplex NPs (PEI-EBV3 and TBP2-EBV3) against an SCC7L tumour model. (**A**) Change in the tumour volume of each mouse within 7 days; (**B**) tumour volume on day 12; (**C**) photos of the ex vivo tumours of each group on day 12; (**D**) tumour inhibition rate; (**E**) H&E-stained sections of tumours in each group. (*n* = 5, one-way ANOVA was for statistical analysis, *** *p* < 0.001, * *p* < 0.05, ns: no significant difference). Bar = 100 μm.

Latent membrane protein 1 (LMP1), encoded by the typical oncogene *Lmp1* of Epstein–Barr virus (EBV) infection (can be detected in both precancerous state and nasopharyngeal carcinoma state), promotes the proliferation and metastasis of nasopharyngeal carcinoma cells by regulating the downstream signalling pathways of p38, NF-κB and PI3K [7,38]. Therefore, we built an LMP1-overexpressed SCC7 cell model (Figure S14) to simulate precancerous lesions/cancer caused by EBV infection and constructed a LMP1-targeting CRISPR/Cas9 system based on the frCas9 system to evaluate the in vivo gene editing ability of TBP2-plasmids polyplex NPs. Figure 9 shows the pharmacodynamic results. The PBS and PEI-EBV3 groups showed a gradual increase in tumour volume, whereas the growth rate of the PEI-EBV3 group was slightly slower than that of the PBS group (Figure 9A–C). The TBP2-EBV3 group exhibited a significant reduction in tumour volume, leading to a smaller tumour volume compared with the other two groups, displaying considerable disparities. Figure 9D displays the rate of tumour inhibition in every group, which was approximately 45% and 25% for the TBP2-EBV3 and PEI-EBV3 groups, respectively. The tumour growth inhibition rate in the TBP2-EBV3 group was significantly higher than that in the PEI-EBV3 group. However, the TBP2-EBV3 group exhibited significant differences compared with the PBS and PEI-EBV3 groups. These results suggest that the TBP2-EBV3 NPs exhibited a more potent inhibitory effect on tumour growth, whereas the PEI-EBV3 NPs demonstrated a minor tumour growth inhibition effect. A possible reason for this phenomena may be critical DNA damage induced by CRISPR/Cas9 system, which can be toxic and can promote cell elimination pathways (i.e., apoptotic and necrotic death) [39]. Owing to its high transfection efficiency, TBP2-EBV3 may cause considerable DNA damage and thus inhibit tumour growth. However, for PEI-EBV3, there was a negligible effect on cell growth. This outcome could be attributed to the relatively weaker gene editing ability of PEI-EBV3 compared to that of TBP2-EBV3. H&E staining (Figure 9E and Figure S14) showed that the tumour cells in the PBS group had large nucleus and clear boundaries, while the TBP2-EBV3 group exhibited local tumour cell shrinkage and abnormal cell morphology compared to the PBS group. Obvious necrosis was also found in the TBP2-EBV3-treated group. The PEI-EBV3 group also showed some degree of tumour cell shrinkage, and parts of tumour cells demonstrated necrosis, but to a lesser extent than that observed in the TBP2-EBV3 group.

**Figure 10:**
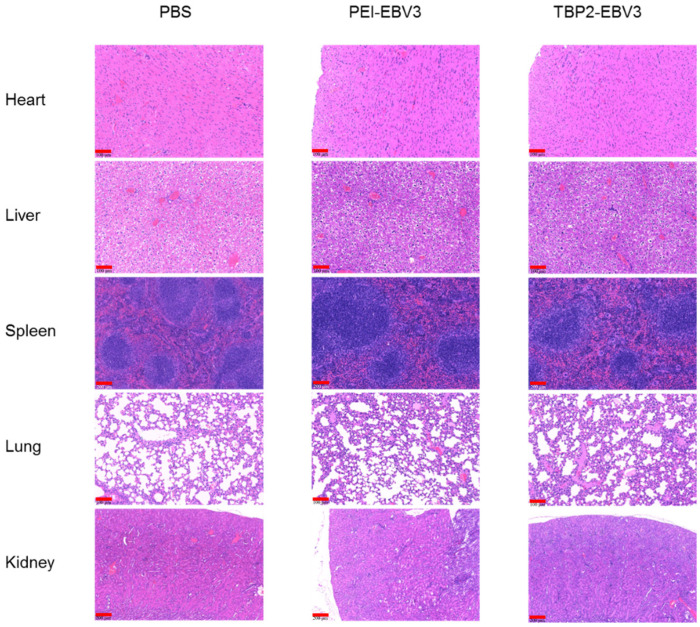
The H&E staining results of major organs after treatment by the polyplex NPs (PEI-EBV3 and TBP2-EBV3) on day 12. Bar = 100 μm.

**Figure 11:**
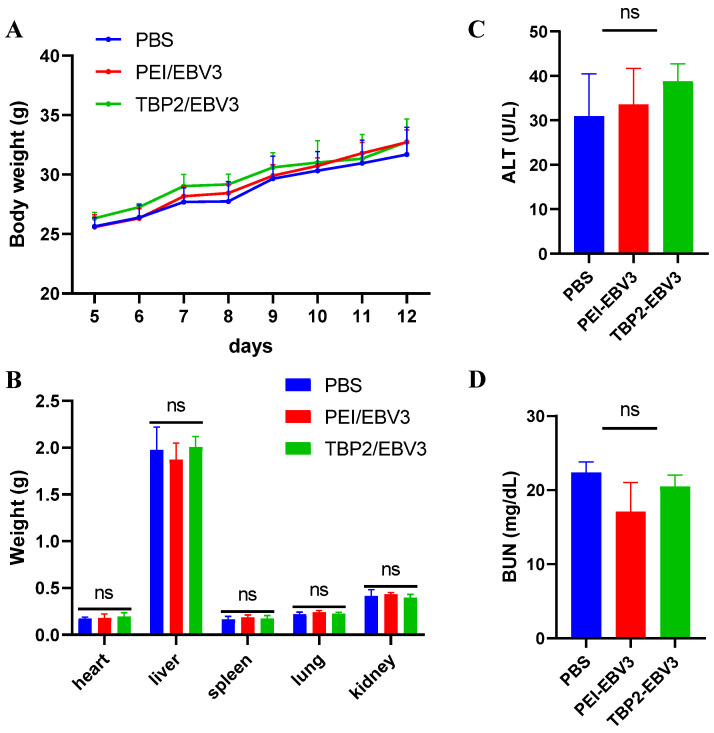
Safety evaluation after treatment by the polyplex NPs (PEI-EBV3 and TBP2-EBV3) on day 12. (**A**) body weight of mice (*n* = 5), (**B**) weight of major organs (*n* = 5), (**C**) ALT (*n* = 3), and (**D**) BUN (*n* = 3). A one-way ANOVA was used for statistical analysis; ns: no significant difference.

A safety evaluation of the drugs in each group of tumour-bearing mice was performed. Figure 10 displays the H&E-stained sections of the major organs for each group, which exhibited normal heart muscle fibres, liver sinusoids, spleen sinusoids, lung alveolar capillaries, and renal glomeruli, with no significant differences between the groups. The weight of each mouse gradually increased within 7 days of injection, with no significant differences observed among the three groups (Figure 11A). The weight of the major organs also showed no significant differences (Figure 11B). Figure 11C,D show the blood biochemical indicators in each group. The typical reference ranges for ALT and BUN in mice are 10.06–96.47 U/L and 10.81–34.74 mg/dL, respectively. The levels of ALT and BUN in each group were within the normal reference range, suggesting that the polyplex NPs did not damage the liver and kidneys. These results provided solid evidence that the gene therapy used in this study was safe and effective.

## 4. Conclusions

In this study, we successfully synthesised three types of TBP polymers with different molecular weights and functional end groups by adjusting the feeding ratio of BDD and AP. TBP2, with a molecular weight of 39,340 Da and the end group of AMP, presented the highest transfection efficiency. TBP2 electrostatically compressed the GFP plasmid into uniformly spherical polyplex NPs with a diameter of 140 nm and a mass ratio of 75:1. The maximum transfection efficiency of the TBP2-GPF polyplex NPs in 293T, HeLa, SiHa, SCC-7, and C666-1 cells was 94.9%, 55.9%, 50.4% 35.5% and 14.4% at TBP2 concentrations of 50, 75, 100, 75 and 75 µg/mL, respectively, with low cytotoxicity (viability > 80%). For Me180 cell line, the viability of cells was approximately 60% with a maximum transfection efficiency of 36.2%, indicating some cationic cytotoxicity. Furthermore, TBP2 efficiently transported the *MUC2*-targeting CRISPR/cas9 gene editing plasmid into 293T cells, resulting in successful gene disruption. This establishes the possibility of using TBP2 in gene therapy in even greater quantities. In KM mice, the NPs formed by TBP2 and corresponding plasmids remained at the tumour site even after injection into the periphery of the tumour, gradually decreasing to a slightly lower level after 24 h. Moreover, these NPs were significantly better than those formed by PEI and its corresponding plasmids. Injection of the TBP2-EBV3 gene editing system every other day in KM mice significantly delayed tumour growth, showing excellent safety and efficacy compared to the PEI-EBV3 group. As a cationic gene delivery system, the application of TBP via intravenous injection is greatly limited. The findings here demonstrate that a better way to use a gene editing system consisting of TBP2 and therapeutic plasmids in gene therapy is via local administration, for example, in cases of cervical cancer and nasopharyngeal carcinoma, wherein therapy can be administered directly through vagina and nasal routes. Further investigation is needed to comprehensively evaluate the in vivo behaviour (for example, mucus penetration and tissue penetration) and application prospects of this system.

## Figures and Tables

**Table 1:** Feed ratio and GPC results of TBP1, TBP2, TBP3.

Molar Ratio	BDD	TPGS-A	AP	Mw (g/mol)	Mn (g/mol)	PDI
TBP1	0.95	0.10	0.90	22,110	17,990	1.23
TBP2	0.95	0.10	1.00	39,340	28,940	1.36
TBP3	0.95	0.10	1.10	40,330	29,960	1.35

## Data Availability

Data are contained within the article and Supplementary Materials.

## Supplementary Materials

The following supporting information can be downloaded at: https://www.mdpi.com/article/10.3390/pharmaceutics16020213/s1, Figure S1: Synthesis and characteristics of TBP; Figure S2: ^1^H-NMR spectrum of TBP1=, TBP2=, TBP3=; Figure S3: ^1^H-NMR spectrum of TBP1, TBP2, TBP3; Figure S4: FT-IR spectrum of TBP1, TBP2, TBP3; Figure S5: GPC chromatograms of TBP1, TBP2, TBP3; Figure S6: Representative cytometric analysis of transfection efficiency of 293T with TBP1, TBP2, and TBP3-GFP polyplex NPs at a mass ratio of 30:1, 50:1, 75:1, 100:1. (UL: GFP positive, LL: GFP negative); Figure S7: DLS results of TBP2-GFP polyplex NPs at different mass ratios; Figure S8: ζ-potential (A) and particle size change (B) of TBP2-GFP and PBAE-GFP polyplex NPs at a mass ratio of 75:1. The data represent the mean ± SD (*n* = 3 per group); Figure S9: IC50 values of TBP2/TBP2-GFP in different cell lines as calculated by MTT assays; Figure S10: Cell viability testing of PBAE-GFP in 293T; Figure S11: Representative cytometric analysis of transfection efficiency of 293T with TBP1, TBP2, and TBP3-GFP polyplex NPs at a mass ratio of 30:1, 50:1, 75:1, 100:1. (UL: GFP positive, LL: GFP negative); Figure S12: The distribution of drug in each group after injection over time (*n* = 3); Figure S13: The fluorescence intensity of major organs in each group at 24 h after injection; Figure S14: Sanger sequencing results of C666-1 and SCC7L1 (C666-1 above and SCC7L below, showing the same gene fragment sequence); Figure S15: The H&E stained sections of tumours and major organs in each group; Table S1: Former and reverse primers of MUC and ODN.
