# Supplementary material for: TPGS-b-PBAE Copolymer-Based Polyplex Nanoparticles for Gene Delivery and Transfection In Vivo and In Vitro
Source: Pharmaceutics. 2024 Jan 31;16(2):213. doi: 10.3390/pharmaceutics16020213 (PMC10891721; doi:10.3390/pharmaceutics16020213)
Supplement: Supplementary file 1 [file pharmaceutics-16-00213-s001.zip › pharmaceutics-2728815-supplementary.pdf]

Supplementary Material:

TPGS-b-PBAE Copolymer-Based Polyplex Nanoparticles for Gene Delivery and Transfection In Vivo and In Vitro

Jiahui Ding [1,†], Handan Zhang [1,†], Tianli Dai [1], Xueqin Gao [2], Zhongyuan Yin [3], Qiong Wang [3], Mengqi Long [4,*] and Songwei Tan [1,*]

[1] School of Pharmacy, Tongji Medical College, Huazhong University of Science and Technology, Wuhan 430030, China; m201975433@alumni.hust.edu.cn (J.D.); m202175567@hust.edu.cn (H.Z.)
[2] Department of Pharmacy, Union Hospital, Tongji Medical College, Huazhong University of Science and Technology, Wuhan 430022, China
[3] Cancer Center, Union Hospital, Tongji Medical College, Huazhong University of Science and Technology, Wuhan 430022, China; wdyxywq@hust.edu.cn (Q.W.)
[4] Department of Otolaryngology, The Fifth Affiliated Hospital of Sun Yat-sen University, Meihua 52nd Road, Xiangzhou District, Zhuhai 510009, China
* Correspondence: longmq5@mail.sysu.edu.cn (M.L.); tansw@hust.edu.cn (S.T.)
† These authors contributed equally to this work.

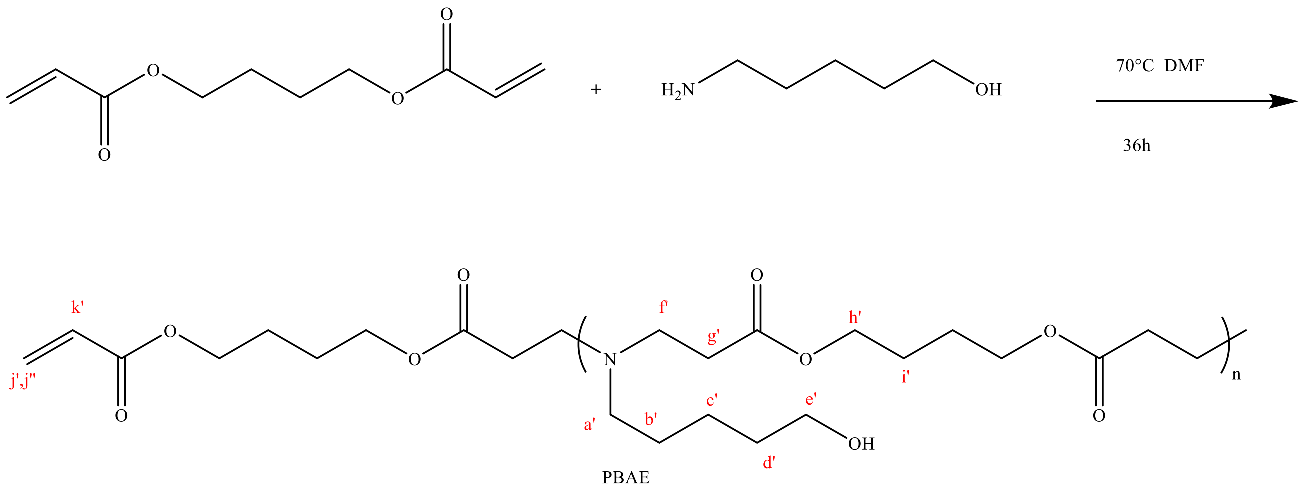

Figure S1. Synthesis of PBAE

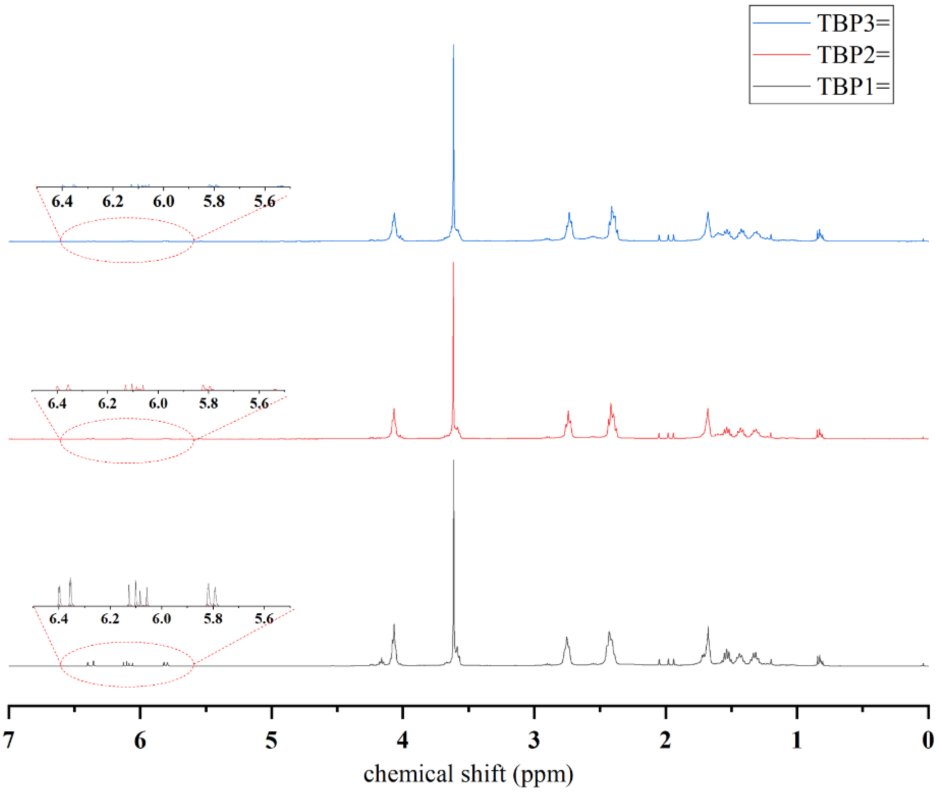

Figure S2. ^{1}H-NMR spectrum of TBP1=, TBP2=, TBP3=

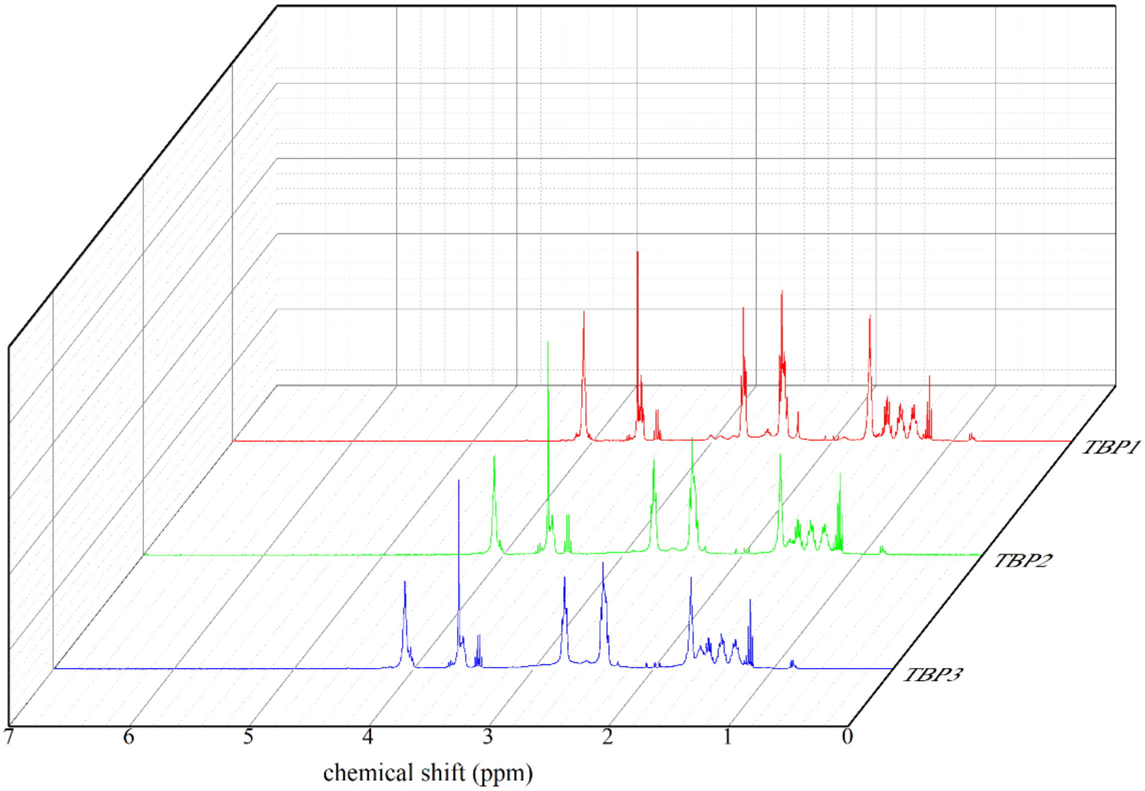

Figure S3. ^{1}H-NMR spectrum of TBP1, TBP2, TBP3

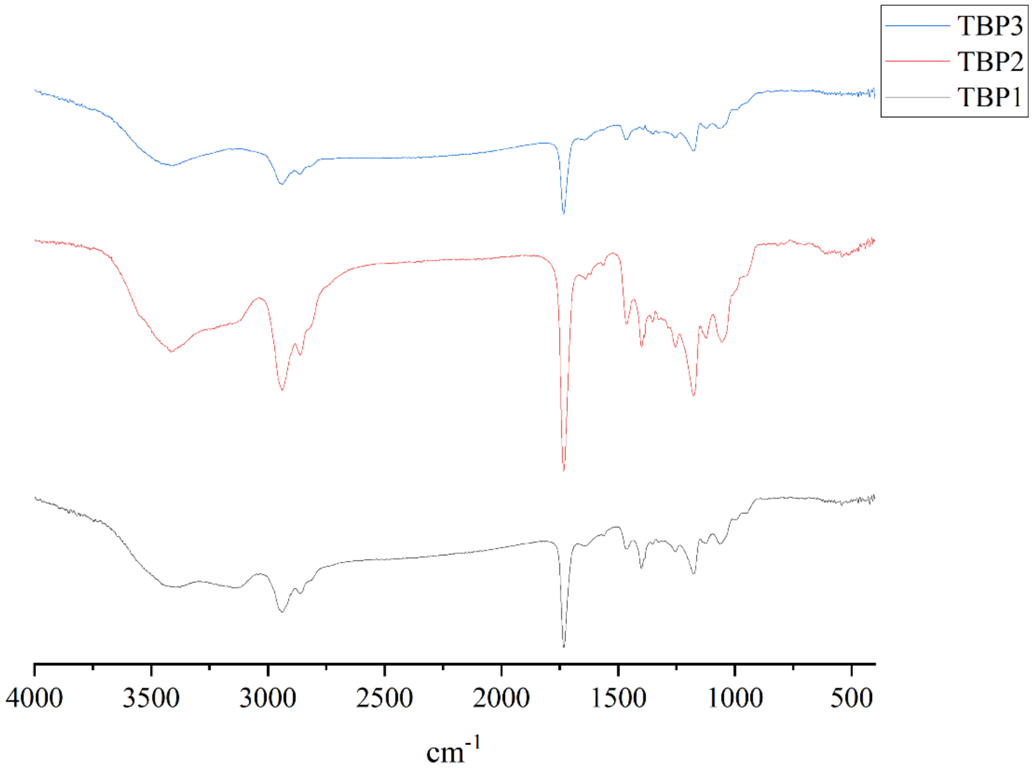

Figure S4. FT-IR spectrum of TBP1, TBP2, TBP3

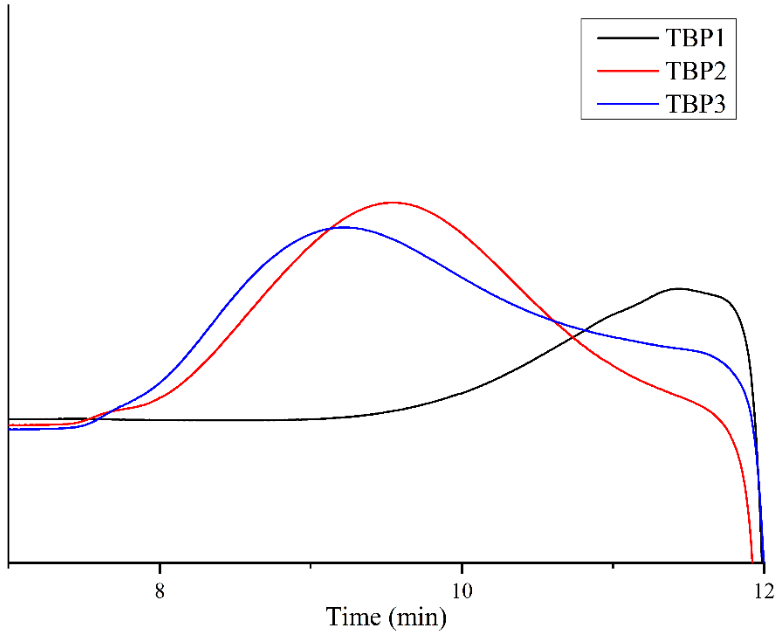

Figure S5. GPC chromatograms of TBP1, TBP2, TBP3

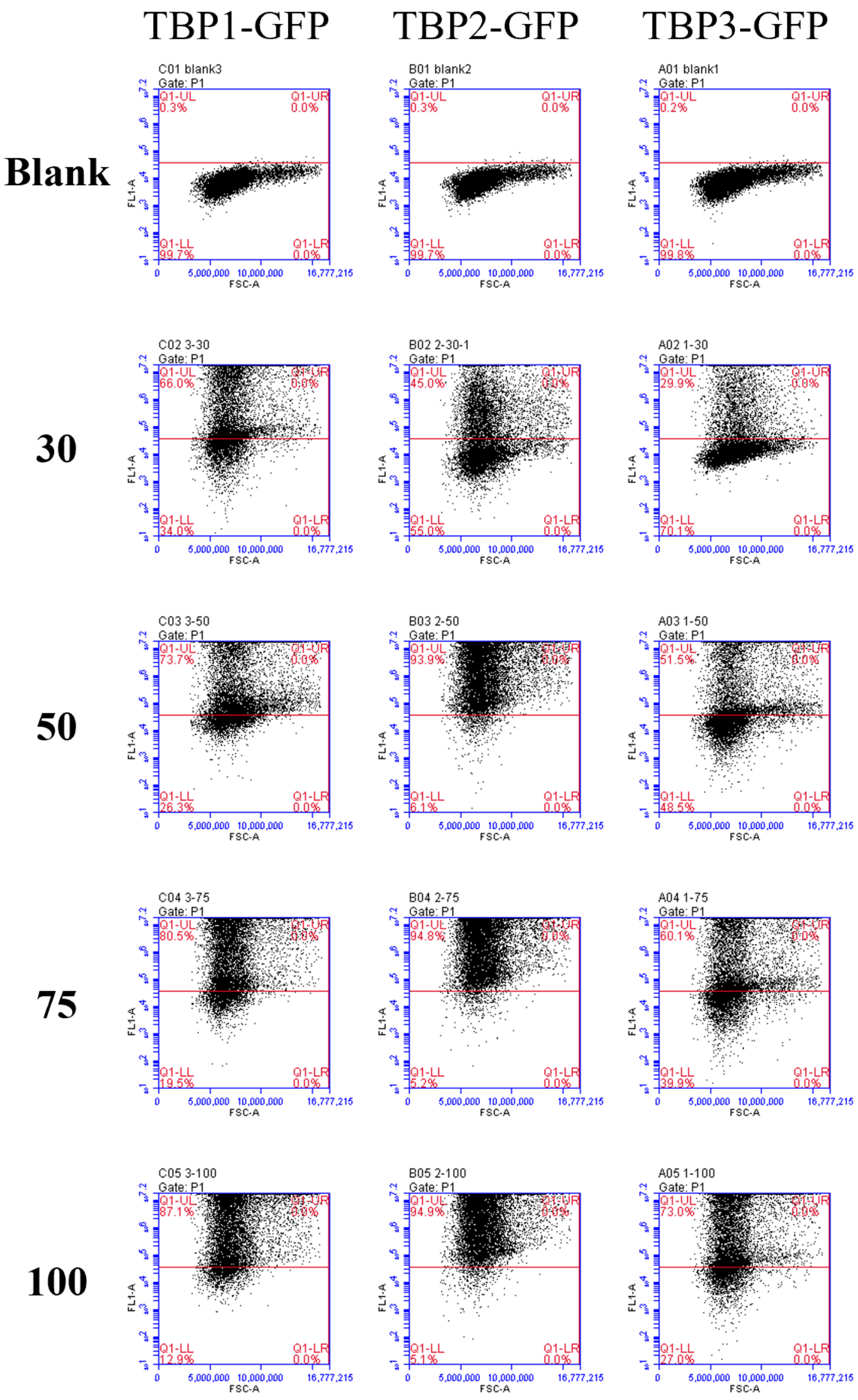

Figure S6. Representative cytometric analysis of transfection efficiency of 293T with TBP1, TBP2, TBP3-GFP polyplex NPs at mass ratio of 30:1, 50:1, 75:1, 100:1. (UL: GFP positive, LL: GFP negative)

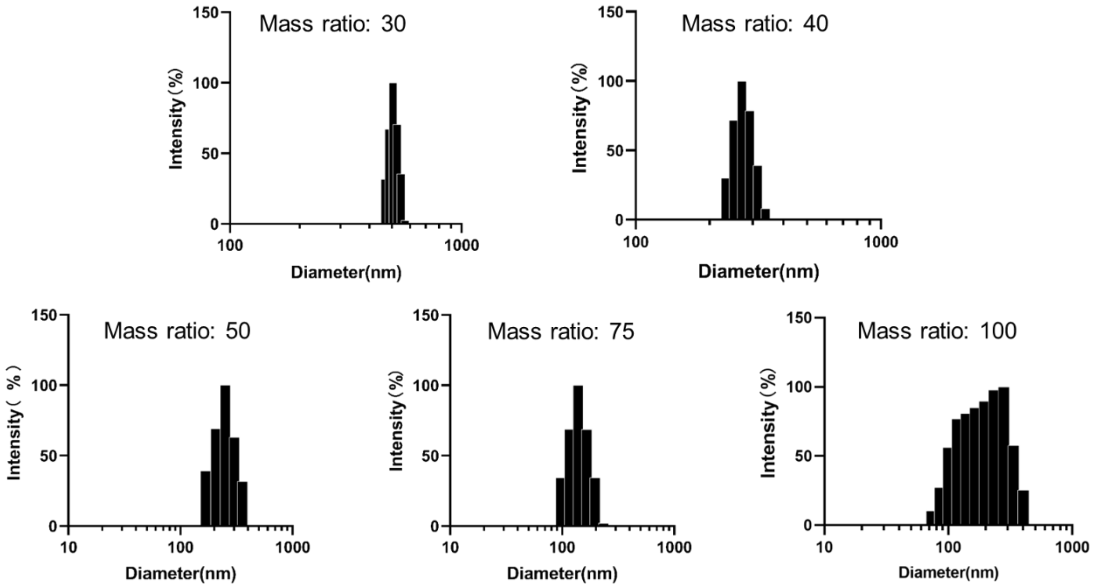

Figure S7. DLS results of TBP2-GFP polyplex NPs at different mass ratios

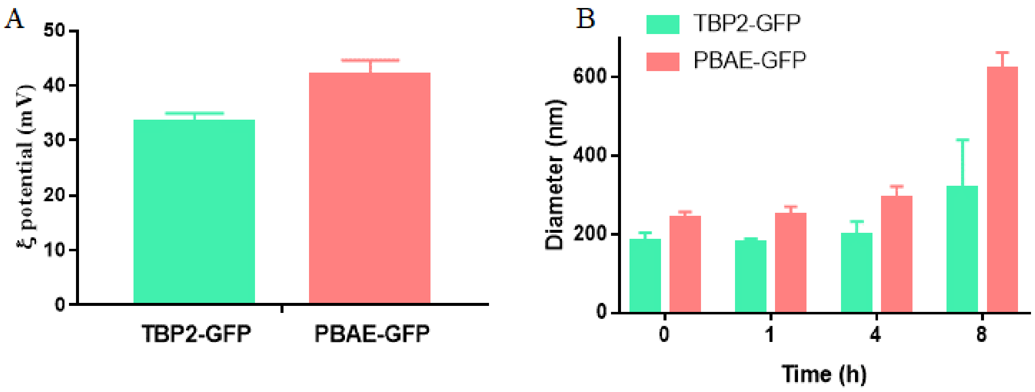

Figure S8. ζ-potential (A) and particle size change (B) of TBP2-GFP and PBAE-GFP polyplex NPs at mass ratio of 75:1 measured by DLS. The data represents the average value of 3 tests.

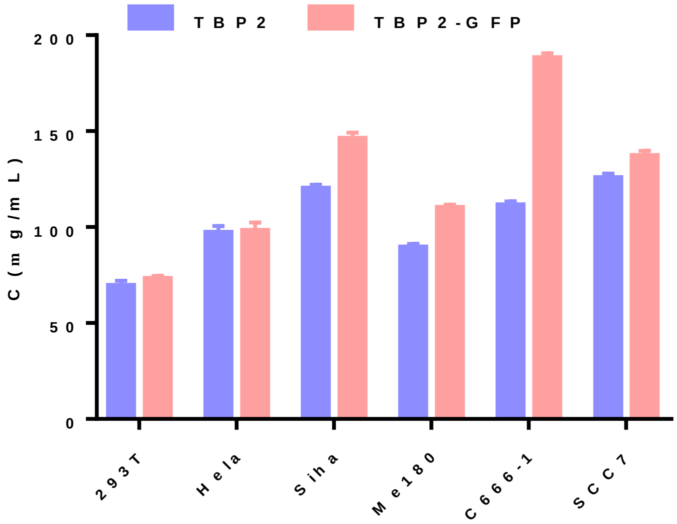

Figure S9. IC50 values of TBP2/TBP2-GFP in different cell lines as calculated by MTT assays.

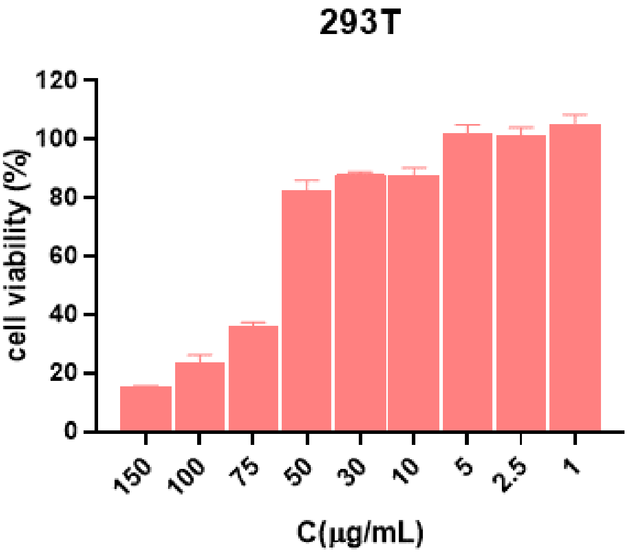

Figure S10. Cell viability testing of PBAE-GFP in 293T

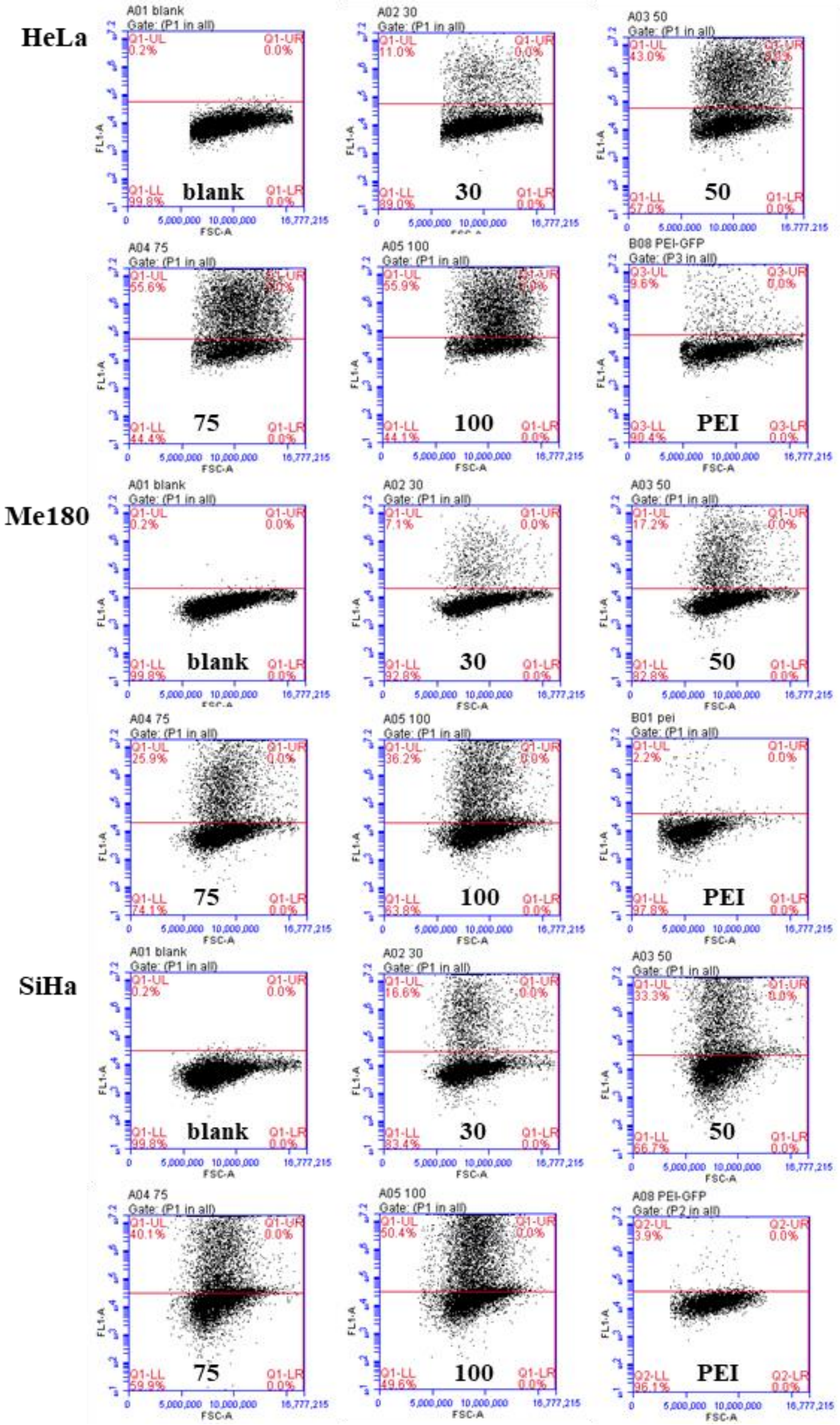

HeLa
A01 blank
Gate: (P1 in all)
Q1-UL 0.2%
Q1-UR 0.0%
Q1-LL 99.8%
Q1-LR 0.0%
blank
A02 30
Gate: (P1 in all)
Q1-UL 11.0%
Q1-UR 0.0%
Q1-LL 89.0%
Q1-LR 0.0%
30
A03 50
Gate: (P1 in all)
Q1-UL 43.0%
Q1-LL 57.0%
Q1-LR 0.0%
50
A04 75
Gate: (P1 in all)
Q1-UL 55.6%
Q1-LL 44.4%
Q1-LR 0.0%
75
A05 100
Gate: (P1 in all)
Q1-UL 55.9%
Q1-LL 44.1%
Q1-LR 0.0%
100
B08 PEI-GFP
Gate: (P3 in all)
Q3-UL 9.6%
Q3-UR 0.0%
Q3-LL 90.4%
Q3-LR 0.0%
PEI
Me180
A01 blank
Gate: (P1 in all)
Q1-UL 0.2%
Q1-UR 0.0%
Q1-LL 99.8%
Q1-LR 0.0%
blank
A02 30
Gate: (P1 in all)
Q1-UL 7.1%
Q1-UR 0.0%
Q1-LL 92.8%
Q1-LR 0.0%
30
A03 50
Gate: (P1 in all)
Q1-UL 17.2%
Q1-UR 0.0%
Q1-LL 82.8%
Q1-LR 0.0%
50
A04 75
Gate: (P1 in all)
Q1-UL 25.9%
Q1-UR 0.0%
Q1-LL 74.1%
Q1-LR 0.0%
75
A05 100
Gate: (P1 in all)
Q1-UL 36.2%
Q1-LL 63.8%
Q1-LR 0.0%
100
B01 pei
Gate: (P1 in all)
Q1-UL 2.2%
Q1-UR 0.0%
Q1-LL 97.8%
Q1-LR 0.0%
PEI
SiHa
A01 blank
Gate: (P1 in all)
Q1-UL 0.2%
Q1-UR 0.0%
Q1-LL 99.8%
Q1-LR 0.0%
blank
A02 30
Gate: (P1 in all)
Q1-UL 16.6%
Q1-UR 0.0%
Q1-LL 83.4%
Q1-LR 0.0%
30
A03 50
Gate: (P1 in all)
Q1-UL 33.3%
Q1-LL 66.7%
Q1-LR 0.0%
50
A04 75
Gate: (P1 in all)
Q1-UL 40.1%
Q1-UR 0.0%
Q1-LL 59.9%
Q1-LR 0.0%
75
A05 100
Gate: (P1 in all)
Q1-UL 50.4%
Q1-UR 0.0%
Q1-LL 49.6%
Q1-LR 0.0%
100
A08 PEI-GFP
Gate: (P2 in all)
Q2-UL 3.9%
Q2-UR 0.0%
Q2-LL 96.1%
Q2-LR 0.0%
PEI
FL1-A
FSC-A
0
5,000,000
10,000,000
16,777,215

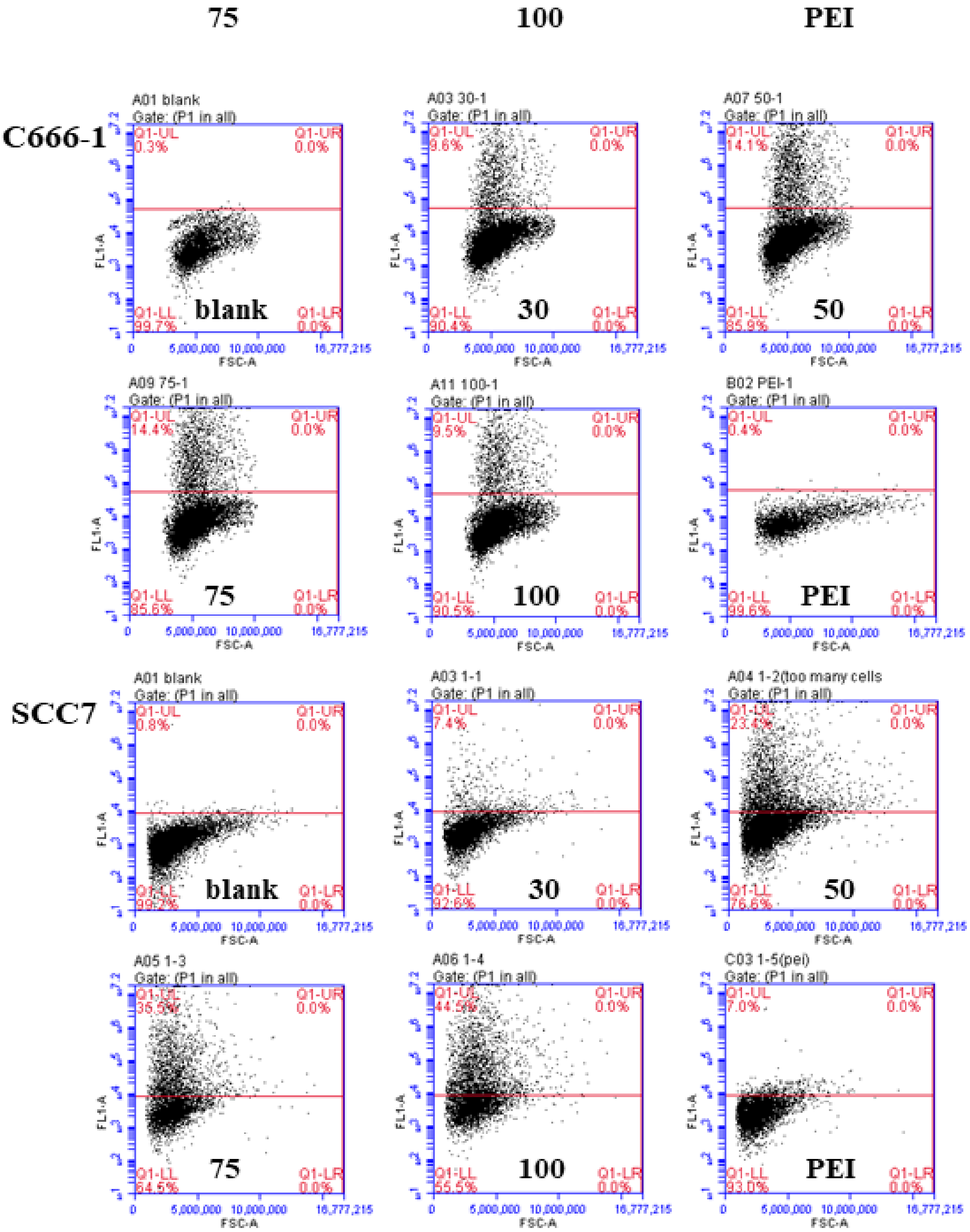

Figure S11. Representative cytometric analysis of transfection efficiency of 293T with TBP1, TBP2, TBP3-GFP polyplex NPs at mass ratio of 30:1, 50:1, 75:1, 100:1. (UL: GFP positive, LL: GFP negative)

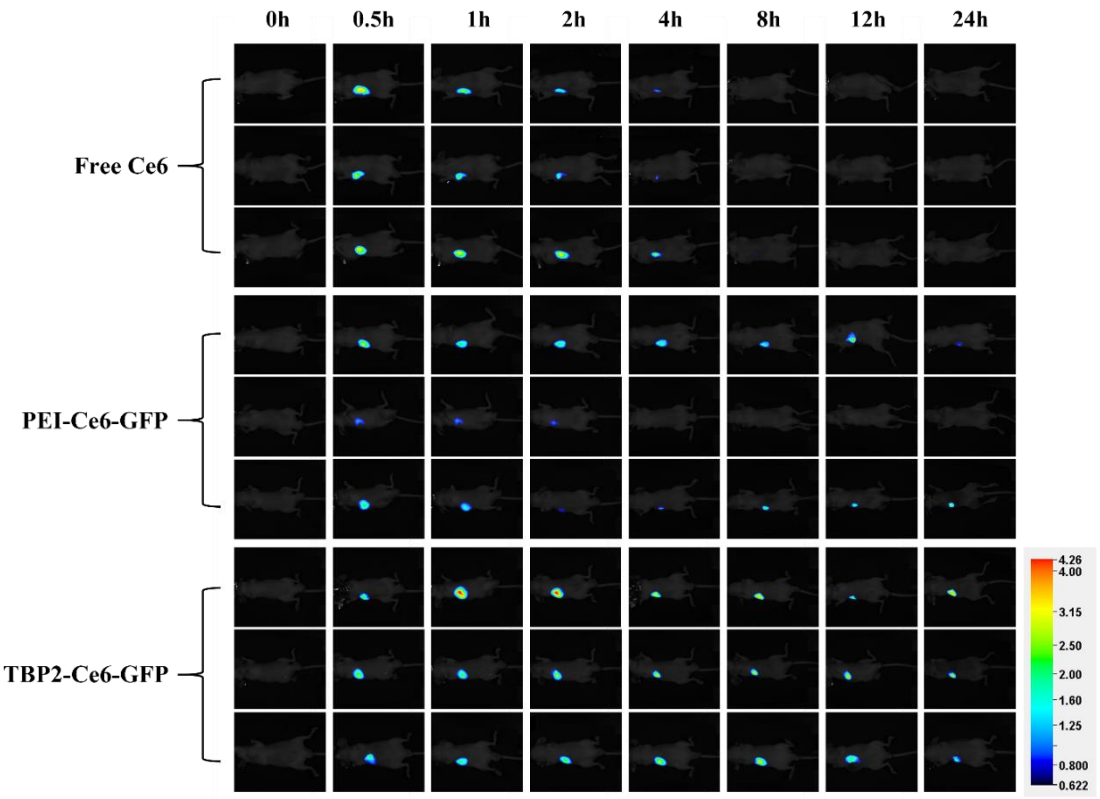

Figure S12. The distribution of drug in each group after injection over time(n=3)

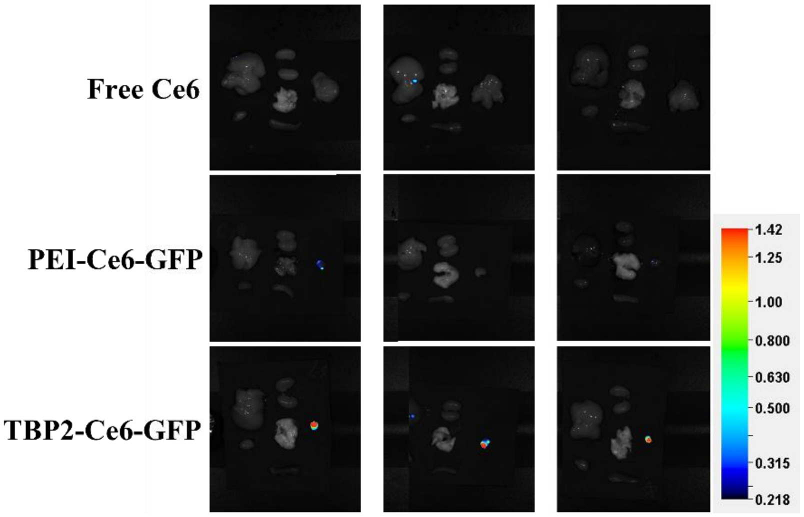

Figure S13. The fluorescence intensity of major organs in each group at 24 hours after injection

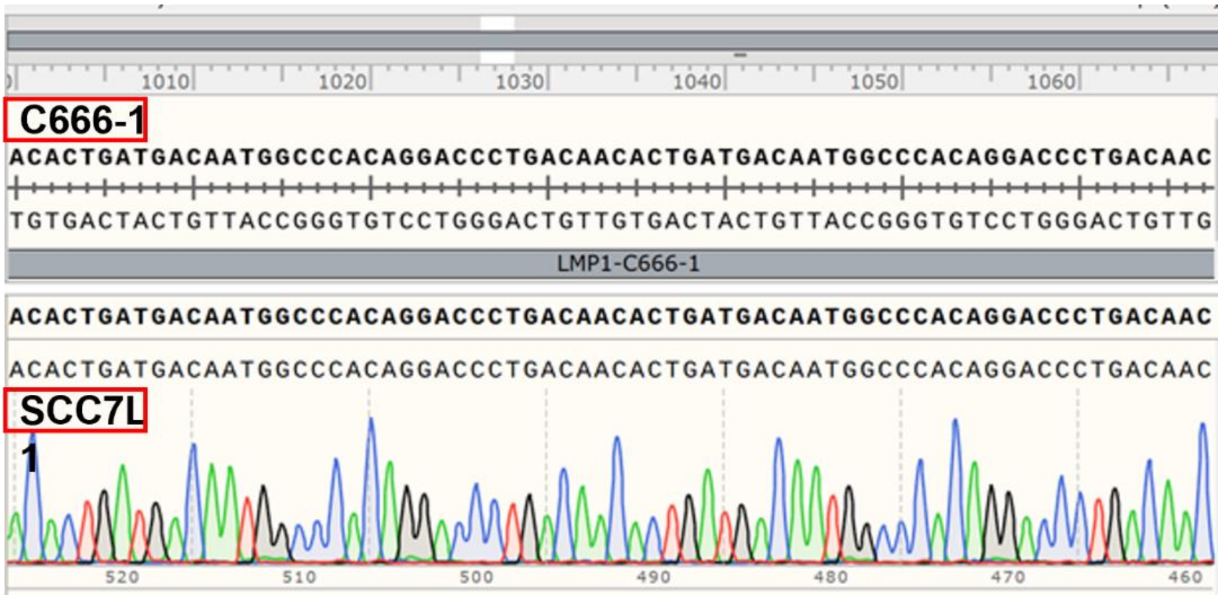

Figure S14. Sanger sequencing results of C666-1 and SCC7L1 (C666-1 above and SCC7L below, showing the same gene fragment sequence).

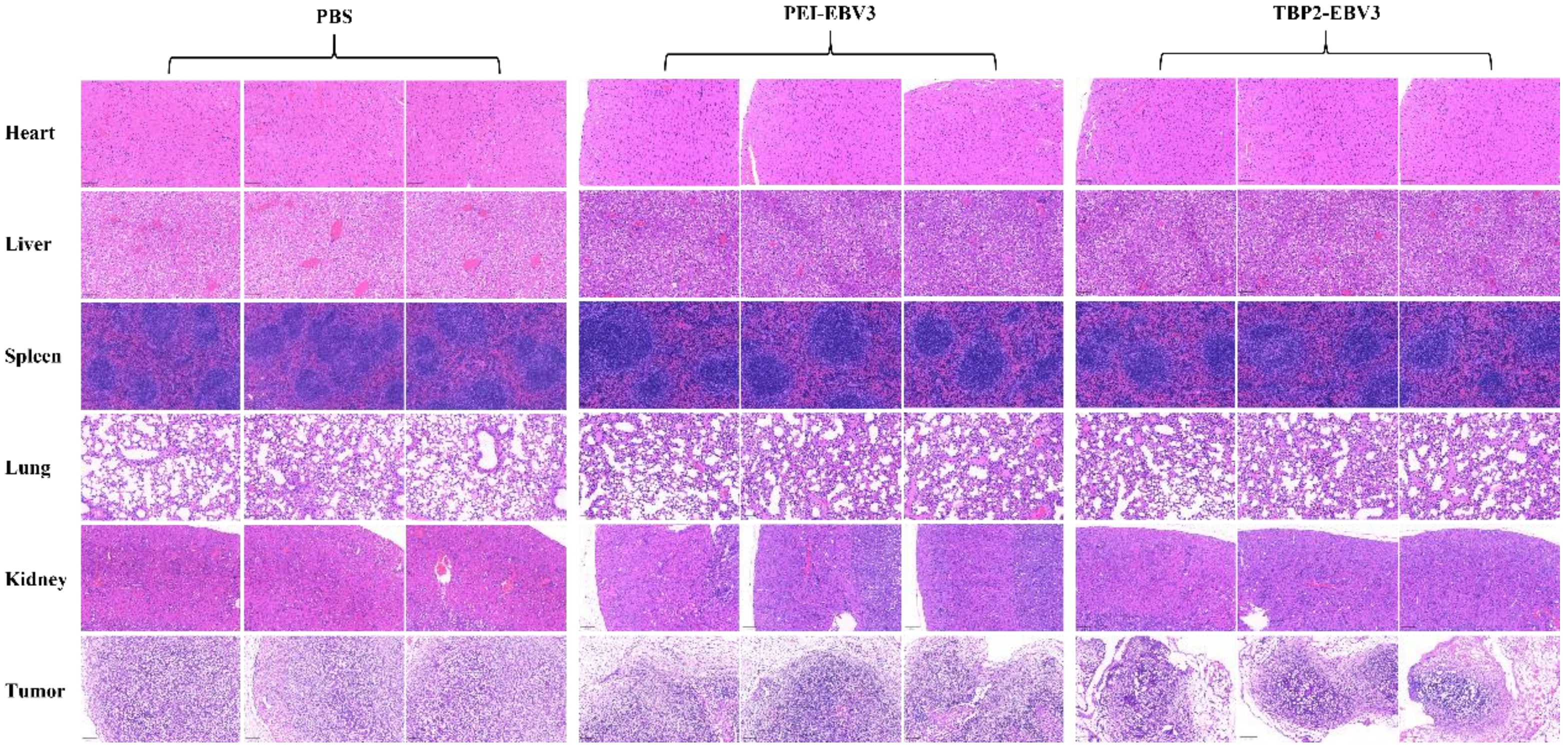

Figure S15. The H&E stained sections of tumors and major organs in each group

Table S1. Former and reverse primers of MUC and ODN

primer	sequence
MUC2-F	CAGCACGTCATCCTGAAGGT
MUC2-R	AAGACACCCTGGAGACACCT
ODN-F	TTGAGTTGTCATATGTTAATAACGGT
ODN-R	ACCGTTATTAACATATGACAACTCAA

ACTGP-9A5BE2F6-BD82435F-D6B74D98-DB8CC766
